# Developing specific molecular biomarkers for thermal stress in salmonids

**DOI:** 10.1186/s12864-018-5108-9

**Published:** 2018-10-16

**Authors:** Arash Akbarzadeh, Oliver P Günther, Aimee Lee Houde, Shaorong Li, Tobi J Ming, Kenneth M Jeffries, Scott G Hinch, Kristina M Miller

**Affiliations:** 1 Fisheries and Oceans Canada, Pacific Biological Station, 3190 Hammond Bay Road, Nanaimo, BC V9T 6N7 Canada; 2 Department of Fisheries, Faculty of Marine Science and technology, University of Hormozgan, P.O. Box: 3995, Bandar Abbas, Iran; 3 Günther Analytics, Vancouver, BC Canada; 4 Department of Biological Sciences, University of Manitoba, Winnipeg, MB R3T 2N2 Canada; 5 Pacific Salmon Ecology and Conservation Laboratory, Department of Forest and Conservation Sciences, University of British Columbia, Vancouver, BC V6T1Z4 Canada

**Keywords:** Gene expression, Salmon FIT-CHIPs, Biomarker, Pacific salmon, Thermal stress

## Abstract

**Background:**

Pacific salmon (*Oncorhynchus spp.*) serve as good biological indicators of the breadth of climate warming effects on fish because their anadromous life cycle exposes them to environmental challenges in both marine and freshwater environments. Our study sought to mine the extensive functional genomic studies in fishes to identify robust thermally-responsive biomarkers that could monitor molecular physiological signatures of chronic thermal stress in fish using non-lethal sampling of gill tissue.

**Results:**

Candidate thermal stress biomarkers for gill tissue were identified using comparisons among microarray datasets produced in the Molecular Genetics Laboratory, Pacific Biological Station, Nanaimo, BC, six external, published microarray studies on chronic and acute temperature stress in salmon, and a comparison of significant genes across published studies in multiple fishes using deep literature mining. Eighty-two microarray features related to 39 unique gene IDs were selected as candidate chronic thermal stress biomarkers. Most of these genes were identified both in the meta-analysis of salmon microarray data and in the literature mining for thermal stress markers in salmonids and other fishes. Quantitative reverse transcription PCR (qRT-PCR) assays for 32 unique genes with good efficiencies across salmon species were developed, and their activity in response to thermally challenged sockeye salmon (*O. nerka*) and Chinook salmon (*O. tshawytscha*) (cool, 13–14 °C and warm temperatures 18–19 °C) over 5–7 days was assessed. Eight genes, including two transcripts of each SERPINH1 and HSP90AA1, FKBP10, MAP3K14, SFRS2, and EEF2 showed strong and robust chronic temperature stress response consistently in the discovery analysis and both sockeye and Chinook salmon validation studies.

**Conclusions:**

The results of both discovery analysis and gene expression showed that a panel of genes involved in chaperoning and protein rescue, oxidative stress, and protein biosynthesis were differentially activated in gill tissue of Pacific salmon in response to elevated temperatures. While individually, some of these biomarkers may also respond to other stressors or biological processes, when expressed in concert, we argue that a biomarker panel comprised of some or all of these genes could provide a reliable means to specifically detect thermal stress in field-caught salmon.

**Electronic supplementary material:**

The online version of this article (10.1186/s12864-018-5108-9) contains supplementary material, which is available to authorized users.

## Background

The physical or abiotic environment consists of many natural factors that can function as stressors either individually or in combination and cause reductions in performance or fitness of living organisms. These include temperature, oxygen, salinity, pH, desiccation and ultraviolet light, as well as anthropogenic factors such as heavy-metals, organic and thermal pollution. Living organisms can be affected by these stressors in two fundamental ways, either by debilitation and ultimately death, or at non-lethal levels of exposure by affecting normal life processes, resulting in ‘capacity’ effects [1, 2]. Recent changes in climate, in particular temperature, have affected ecological processes across a broad range of taxa [3, 4]. Environmental temperatures have extensive biological implications for all organisms, but ectotherms in aquatic systems are particularly affected by thermal profiles and climate regimes in terms of their distribution, physiology and behaviour [5]. Temperature has a fundamental impact on the physiology and biochemistry of individuals, especially ectothermic fish that inhabit thermally variable environments [6, 7]. Increases in water temperature can occur due to a variety of natural and anthropogenic causes [8]. Many fish populations can be acutely and chronically exposed to water temperatures outside their species- or population-specific preferred thermal ranges, and these occurrences are expected to become more frequent due to anthropogenic climate warming [9]. Such temperature changes have the potential to disrupt ecosystem stability resulting in species-specific differences in susceptibility to factors such as physiological tolerance range, potential for adaptation to abiotic stressors, and life history characteristics, all playing important roles in determining climate change ‘winners’ versus ‘losers’ status [10].

To compensate for the effects of temperature changes, ectotherms can respond to chronic temperature changes by increasing the quantity of tissue or enzyme needed for different physiological tasks, or by expressing protein isoforms that are more appropriate for the new thermal conditions. On the other hand, proteins that are needed in lesser amounts in the new thermal regime could be depressed or downregulated. Although expression of proteins can be changed by multiple mechanisms during synthesis and degradation, temperature dependent changes in transcription of genes are one of the key events in modifying the proteome of the tissues [11]. The expression of mRNA represents a primary response to environmental change that often, but not always, leads to changes in protein expression and cell function [12]. Stress responses involve expression of a series of evolutionarily conserved stress-responsive genes that include genes controlling cell cycle, protein folding and repair, DNA and chromatin stabilization and repair, removal of damaged proteins, and energy metabolism [13]. Therefore, understanding how contributing cellular and molecular processes shape organisms’ abilities to cope with thermal regime shifts is critical to forecasting species’ responses to climate warming [14–16].

Transcriptional analysis is an important means for investigating the physiological response to environmental changes of non-model organisms [17] and species of conservation concern [18]. Using genomic tools to study the potential or candidate genes associated with stress responses, unique signatures or imprints of specific stressors can be resolved that may, in the future, be applied to recognize early signs of stressors present, but not necessarily when there is a direct measurement of those stressors [19]. While some biomarkers are associated with a broad range of stressors, e.g. heat shock proteins and cortisol, different individual stressors also elicit more specific responses through the stimulation of different biological pathways. For example, high water temperature stress enhances the expression of molecular chaperones, heat shock proteins, and genes involved in RNA stabilization, transcriptional regulation, and immunity, as well as genes that are also stimulated under oxidative stress and important in ion homeostasis, and causes shifts in the expression of genes involved in protein biosynthesis and metabolism [9].

Transcriptomic responses can be readily measured using quantitative reverse transcription PCR (qRT-PCR), microarrays, and RNA-sequencing (RNA-seq), the method of choice depending upon the number of markers and individuals to be assessed. RNA-seq is growing in popularity because it can measure the activity of all genes, depending on the depth of sequencing, but is not always cost effective to assess individual variation over dozens to hundreds of samples. While individual qRT-PCR can be used to measure the transcriptional activity of single genes (biomarkers), if dozens to hundreds of biomarkers are required, it is cost and time prohibitive, and requires relatively large tissue samples. There are now a number of high-throughput qRT-PCR platforms available to simultaneously assess dozens of assays across dozens of samples. The microfluidics-based Fluidigm BioMark™ platform, capable of performing 96 assays in 96 samples at once, has been applied extensively in salmon research, often merging high-throughput host biomarker and infectious agent monitoring [20–23]. One key advantage of this platform over others is its flexibility, as assays are loaded onto dynamic arrays at the time they are run rather than coming pre-loaded as is the case for most platforms. As such, one can readily reconfigure the assays utilized across chips, making it easy to customize applications towards specific needs.

Temperature is one of the most important environmental influences on salmonid biology. Temperature influences growth, feeding, metabolism, development of embryos and alevins, timing of life history events such as upstream migration, spawning, freshwater rearing, and seaward migration, and the availability of food [24]. Temperatures at sub-lethal levels can effectively block migration, lead to reduced growth, stress fish, increased susceptibility to infectious disease, affect reproduction and smoltification, and alter competitive dominance [25]. Further, the stressful impacts of water temperatures on salmonids are cumulative and positively correlated to the duration and severity of exposure. The longer the salmonid is exposed to thermal stress, the less chance it has for long-term survival [26]. Pacific salmon (*Oncorhynchus* spp.) serve as good biological indicators of the breadth of climate warming effects on fish because their anadromous life cycle exposes them to environmental challenges in both marine and freshwater environments [27]. It has been suggested that the abundance of Pacific salmon may decline if water temperatures continue to rise as predicted by climate models and particularly because of warming river temperatures during spawning migrations [4]. Indeed, warm temperatures during freshwater spawning migrations in recent years have been associated with high mortality rates in some stocks across the Northeast Pacific [4].

Functional genomics research undertaken by Fisheries and Oceans Canada (DFO), over the past decade has provided physiological insight into factors contributing to reduced salmon survival, especially during critical periods as salmon migrate between freshwater and saltwater environments [9, 28–31]. Migratory salmon are exposed to a multitude of stressors along their migration path that vary by environment and life-history stage, and we expect that both the manifestation of the stress response and its physiological consequence may also vary by these same factors. Thermal stress has been a repeated theme in much of our research, which has explored the cumulative or synergistic impacts of thermal and fisheries-related handling stress, and associations with enhanced pathogen virulence, reduced energy reserves, and reduced ability to maintain homeostasis. While our initial research relied on discovery-based microarray studies, an additional goal was to develop a high throughput biomarker-based tool that could be applied across 100’s to 1000’s of fish to simultaneously monitor for the presence of a number of key stressors and diseases, including thermal stress, using non-destructively sampling of gill tissue, a multifunctional organ which directly contacts the external environment. In order to identify suites of biomarkers specific to different stressors and disease states, we mined microarray data from published challenge studies and the DFO salmon microarray database encompassing more than 4900 microarrays and three salmon species including Chinook (*O. tshawytscha*), sockeye (*O. nerka*), and coho (*O. kisutch*), using both supervised and unsupervised analytical approaches. We also applied text mining on abstracts and full papers for key protein (biomarkers) word associations that have been linked to specific stressors and disease states. Herein, we focus on the biomarkers resolved using this approach for thermal stress in salmon, and present the validation results for the selected biomarker panel on different species of salmonids under both artificial and naturally-induced thermal stress. Our broad objective was to discover a co-expressed biomarker panel of up to 12 genes that together was predictive of the presence of thermal stress, similar to what we have recently published for viral disease diagnostics [22]. We eventually aim to combine individual panels specific to thermal, salinity, and osmotic stress with those predictive of viral, bacterial, and parasitic disease states into a single tool (Salmon FIT-CHIPs) applied using the BioMark platform. Application of this tool would enable researchers and fisheries managers to predict and potentially mitigate the manifestation of stress both in cultured and naturally migrating salmon and develop strategies for improving performance and aid in predictions of marine and return-migration survival.

## Results

### Identification of candidate thermal stress biomarkers

**Fig. 1:**
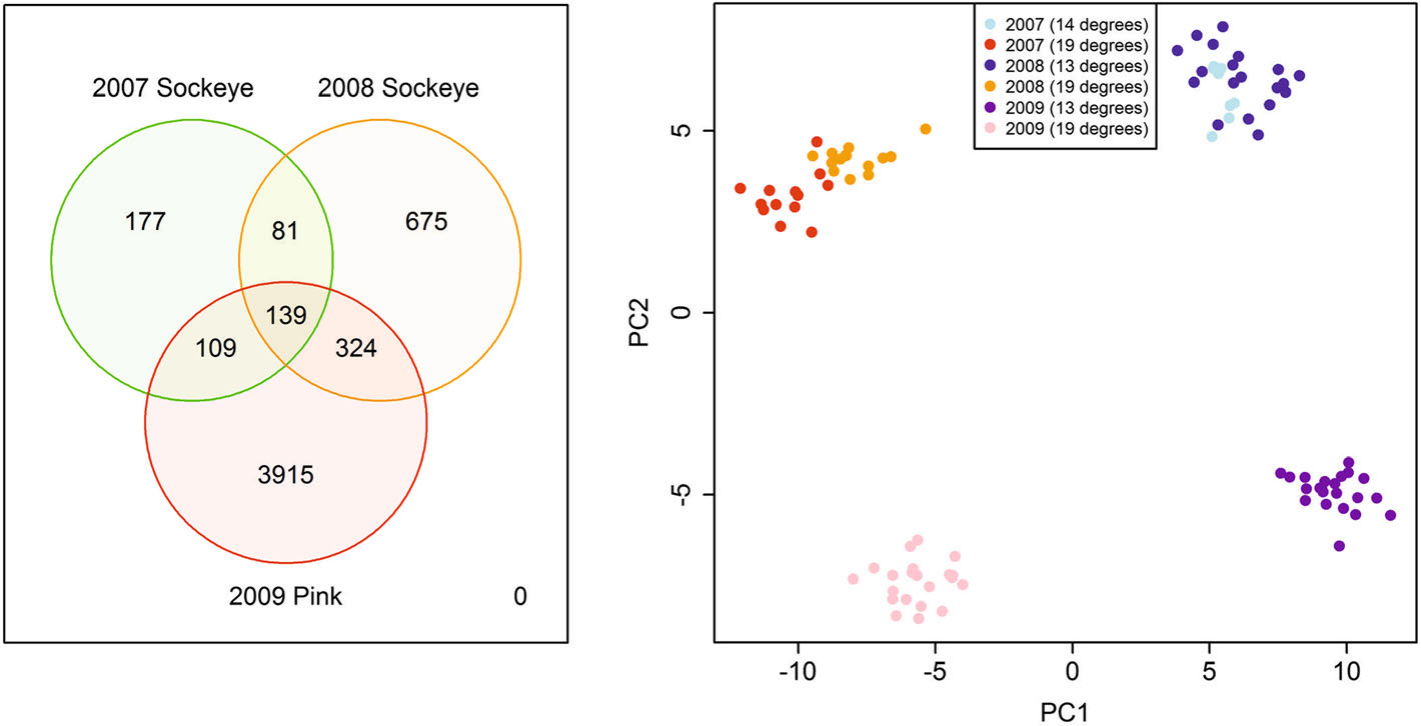
The Venn diagram on the left compares three signatures that were returned by robust limma analysis with an FDR threshold of 0.01 for the 2007 sockeye, 2008 sockeye and 2009 pink salmon data sets (corresponding to signatures DS0101, DS0102 and DS0103 in Table 6). The right plot shows PCA results for 98 samples and 139 EST identifiers returned as significant by all three robust limma analyses

The discovery analysis included a set of three separate analyses of the 2007 sockeye (21 samples), 2008 sockeye (33 samples) and 2009 pink (44 samples) salmon thermal challenge studies with robust limma (FDR < 0.01), and a 3-set comparison of significant genes and fold changes. There were 506 and 1219 significant EST identifiers identified in the 2007 and 2008 sockeye salmon data respectively, and 4487 significant EST identifiers in the 2009 pink salmon data (Fig. 1).

The intersection of the three robust limma analyses returned 139 EST identifiers, which all showed consistent fold change direction across the 3 years. 45 (92%) of 49 identifiers described in Jeffries et al. [9] were included in this set (the four missing identifiers represent two genes: Cytochrome c oxidase subunit VIb isoform 1 and Zinc finger RAD18 domain-containing protein C1orf124) (Additional file 1: Table S1). These analyses were based on the same 98 samples (54 sockeye and 44 pink salmon samples) that were used in Jeffries et al. [9]. Fold change direction of the 139 EST identifiers were 100% concordant across years with 69 identifiers displaying higher and 70 identifiers displaying lower expression in the warmer temperature samples (Additional file 1: Table S1). The five identifiers coding for serpin H1 precursor (SERPINH1) showed the highest fold changes in each year (higher expression in warmer temperatures) with fold changes for 2007 and 2008 sockeye salmon displaying greater values than corresponding fold changes for pink salmon (2009). Several EST identifiers coding for heat shock protein HSP 90 (HSP90AB1) showed higher gene expression in warmer temperatures, with 2008 sockeye salmon showing lower fold changes than 2007 sockeye and 2009 pink salmon.

The union of EST identifiers from the three robust limma analyses (FDR < 0.01) in the Venn diagram in Fig. 1 contains 5420 identifiers that define signature CS0101u. Just nine of these identifiers were selected by the Gene Shaving unsupervised clustering method when applied to the subset of 5254 identifiers in the 3-year MGL temperature data (Additional file 2: Figure S1). All nine identifiers are included in the set of 139 common identifiers from intersection of robust limma analyses (FDR < 0.01) for the 2007, 2008 and 2009 discovery analysis (CS0101i), and are also found in the 49-feature published signature in Jeffries et al. 2014b (ES0013). The 9 identifiers map to SERPINH1 (5 identifiers), cold-inducible RNA-binding protein (CIRBP), splicing factor, arginine/serine-rich 9 (SFRS9) (2 identifiers), and splicing factor, arginine/serine-rich 2 (SFRS2).

Based on 139 EST identifiers in signature CS0101i, the PCA plot shows the projection of the 98 samples onto PC2 vs PC1 (Fig. 1). There is clearly a separation of low vs. high temperature samples along PC1 independent of species while PC2 separates the two species with sockeye salmon showing positive, and pink salmon showing negative PC2 values. Unsupervised analysis methods were also applied to the 2007, 2008 and 2009 data sets separately, and provided additional candidate signatures. In each case, PCA, sparse PCA and gene shaving were applied and the signatures showing the strongest separation of samples by temperature treatment were chosen. For the 2009 pink salmon thermal challenge data this lead to the selection of a 21-feature Gene Shaving signature (signature EX0101a; Additional file 3: Figure S2) that showed a complete and pronounced separation of samples by thermal challenge, and included all 9 EST identifiers from the 3-year MGL temperature analysis described above. An additional 8 of the 21 identifiers were also found in the robust limma combined temperature signature (CS0101i), and 14 of these overlapping identifiers were found in the corresponding 49-identifier signature in Jeffries et al. 2014b [9] (ES0013). Gene shaving applied to the 2007 sockeye salmon data resolved 98 EST identifiers (EX0102a) which also showed a complete and pronounced separation among temperature treatments (Additional file 4: Figure S3). Unsupervised analysis of the MGL 2008 sockeye salmon data revealed that sparse PCA identifier sets on dimension 2 (sPC2) and dimension 3 (sPC3) displayed the clearest resolution by temperature in scatter plots of the first five sparse principal components. The combined 200-identifier sPC2 + sPC3 signature (EX0103a) had several identifiers in common with the 2009 pink salmon signature (EX0101a), the 2007 sockeye salmon signature (EX0102a) (Additional file 5: Figure S4), as well as with the 139-identifier chronic temperature stress response signature (CS0101i), and the 49-identifier signature published in Jeffries et al. [9] (ES0013). The overlap of the five signatures is summarized in Additional file 6: Table S2.

Concordance of several of the discovered and published signatures on chronic and acute temperature stress on 16 K, 32 K and 44 K GRASP platforms (using Blast search-based mapping) are shown in Table 1. Quinn et al. [32] and Jeffries et al. [30] provided chronic temperature stress signatures while the Quinn et al. [33], Lewis et al. [34] and Anttila et al. [12] studies provided acute temperature stress signatures. There are more features in common between the Quinn et al. [32] (chronic) and Anttila et al. [12] (chronic) signatures (17 EST identifiers) than between the Quinn et al. [33] (acute) and Anttila et al. [12] (chronic) signatures (11 EST identifiers). DS0104 is a discovery signature based on robust limma applied to the 18-sample Arctic charr data set used in Quinn et al. [33] (cGRASP32K). The analysis returned a 149-EST identifiers signature, of which more than 50% were found in the ES0023 signature (as defined in Table S2 in Quinn et al. [33] publication). The chronic temperature stress response signatures have consistently more overlap with Quinn et al. [32] (chronic) than with Quinn et al. [33] (acute) signature. There is little overlap with the discovered signature DS0104 (acute) and with the Lewis et al. [34] signatures but more with the 32-feature Jeffries et al. [30] signature.

**Table 1:**
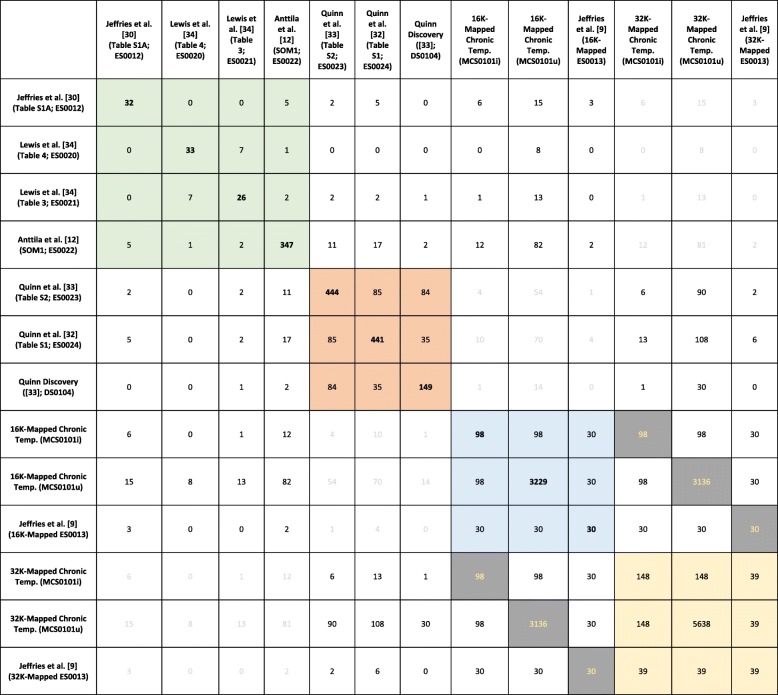
The overlap of features between different temperature stress response signatures. Cells colored in orange (Quinn et al. [32, 33] related signatures) indicate that the corresponding signature was derived on cGRASP32K, i.e. only a subset of features could be tested for in the GRASP16K published signatures (green block). The chronic temperature stress response signatures were based on the cGRASP44K platform. 44 K-to-16 K and 44 K-to-32 K mapping tables were used to map the signatures to the GRASP16K (green) and cGRASP32K (orange) platform. Cells with numbers displayed in light grey color indicate non-optimal comparisons (e.g. 32 K with 44 K-to-16 K) while grey cells with yellow numbers show that some of the mapped 44 K–16 K features were not found by the 44 K–32 K mapping

The 44 K-to-16 K mapping of the 139-EST identifier chronic temperature stress response signature (CS0101i) derived from the MGL 2007–2009 Temperature data set returned 98 EST identifiers while the corresponding 44 K-to-32 K mapping of the same signature returned 148 EST identifiers.

The Rebl et al. [6] study was based on a 4x44K Agilent platform but no data was deposited into GEO. Genes identified in Table 2 in that publication included CIRBP, SERPINH1, SFRS2 and SRSF9, which were consistently found to be important in chronic temperature response in our discovery analysis.

Our results showed that there was an impressive amount of overlap between the signatures derived from different species (sockeye and pink salmon) and different years in the MGL 2007–2009 Temperature data, and the 139-feature CS0101i signature appears to be a strong and robust chronic temperature stress response signature. This signature has large overlap with the EST identifiers in Additional file 1: Table S1 that were derived from unsupervised exploratory analyses of the sockeye and pink salmon data sets. Additional file 6: Table S2 combines signatures derived from supervised and unsupervised analyses of the MGL 2007–2009 Temperature data set to define candidate temperature biomarker signature CTS001.

### Selected candidate thermal stress biomarkers

Eighty-two microarray features related to 39 unique gene IDs were selected as candidate chronic thermal stress biomarkers. Most of these genes were identified both in the meta-analysis of salmon microarray data (Table 3) and in the literature mining for thermal stress markers in salmonids and other fish species (Table 2); many were also known to be involved in pathways related to thermal stress. Some additional EST identifiers selected in our analyses contained no sequence information in Pacific salmon, hence were dropped. All of the selected biomarkers were also included in the signature intersection between robust limma analysis based on the 2007, 2008 and 2009 MGL Temperature data set, except EST identifiers that mapped to heat shock protein 70 (HSP70) and heat shock cognate 70 protein (HSC71). These identifiers were included in the signature EX0102a derived from the 2007 sockeye salmon data. Furthermore, virtually all selected biomarkers were observed in at least 2 of the 11 signatures and studies (Table 2), except of protein disulfide-isomerase A4 precursor (PDIA4). The selected candidate thermal stress biomarkers represented 10 different functional groups including protein folding and rescue, transcription/translation/protein transport, metabolic processes, oxidative stress/ion binding/signal transduction, transmembrane transport, DNA repair, cell structure, protein transporter activity, co-repressor of transcription, growth regulation, and cell cycle and apoptosis (Table 3).

**Table 2:** Selected candidate genes specified to be induced in different tissues during thermal stress in different fish species (Literature references for thermal stress)

Gene	Functional group	Study (tissue and species)
SERPINH1	Upregulation	Gill *O. nerka* (Jeffries et al. [9, 30]), *O. gorbuscha* (Jeffries et al. [9]), *O. mykiss* (Rebl et al. [6]; Wang et al. [7]), *O. tshawytscha* (Tomalty et al. 17]), *Hypomesus transpacificus* (Komoroske et al. [16]) Liver *Gadus morhua* (Hori et al. [46]), *O. mykiss* (Wang et al. [7]), *Puntius sophore* (Mahanty et al. [48]) Muscle *G. morhua* (Hori et al. [46]) Kidney *G. morhua* (Hori et al. [46]), *O. mykiss* (Verleih et al. [45]; Wang et al. [7])
HSP90	Upregulation	Gill *Gillichthys mirabilis* (Buckley et al. [64]; Logan & Somero, [59, 62]), *O. nerka* (Jeffries et al. [30]; Jeffries et al. [9]), *O. gorbuscha* (Jeffries et al. [9]), *Salvelinus alpinus* (Quinn et al. [33]), *Fundulus heteroclitus* (Healy et al. [56]), *H. transpacificus* (Komoroske et al. [16]), *Ictalurus punctatus* (Liu et al. [13]), *O. mykiss* (Rebl et al. [6]), *O. tshawytscha* (Tomalty et al. [17]) Liver *Aphanius ginaunis* (Akbarzadeh & Leder, [96]), *Austrofundulus limnaeus* (Podrabsky & Somero, [81]), *G. morhua* (Hori et al. [46]), *Salmo salar* (Olsvic et al. [97]), *Melanotaenia duboulayi* (Smith et al. [98]), *I. punctatus* (Liu et al. [13]) Cardiac *O. nerka* (Anttila et al. [12]), *O. mykiss* (Vornanen et al. [11]) Muscle *G. mirabilis* (Buckley et al. [64]), *G. morhua* (Hori et al. [46]), *F. heteroclitus* (Healy et al. [56]), *Lates calcarifer* (Newton et al. [99]) Kidney *G. morhua* (Hori et al. [46]), *O. mykiss* (Verleih et al. [45]) Red blood cells *O. mykiss* (Lewis et al. [34]) Testis *A. ginaunis* (Akbarzadeh & Leder, [96])
HSP70/ HSC71	Upregulation	Gill *G. mirabilis* (Buckley et al. [64]; Logan & Somero, [62]), *S. alpinus* (Quinn et al. [33]), *O. mykiss* (Rebl et al. [6]), *F. heteroclitus* (Healy et al. [56]), *H. transpacificus* (Komoroske et al. [16]), *I. punctatus* (Liu et al. [13]), *O. tshawytscha* (Tomalty et al. [17]) Liver *A. ginaunis* (Akbarzadeh & Leder, [96]), *A. limnaeus* (Podrabsky & Somero, [81]), *G. morhua* (Hori et al. [46]), *M. duboulayi* (Smith et al. [98]), *I. punctatus* (Liu et al. [13]) Cardiac *O. nerka* (Anttila et al. [12]), *S. salar* (Lund et al. [100]) Muscle *G. morhua* (Hori et al. [46]) Kidney *G. morhua* (Hori et al. [46]), *O. mykiss* (Verleih et al. [45]) Red blood cells *O. mykiss* (Lewis et al. [34]) Testis *A. ginaunis* (Akbarzadeh & Leder, [96])
PDIA4	Upregulation	Gill *O. nerka* (Jeffries et al. [30]), *O. tshawytscha* (Tomalty et al. [17]), *G. mirabilis* (Buckley et al. [64]; Logan & Somero, [62]), *Trematomus bernacchii* (Buckley & Somero, [65]), *I. punctatus* (Liu et al. [13]) Liver *I. punctatus* (Liu et al. [13])
SEPW1	Upregulation	Gill *O. nerka* & *O. gorbuscha* (Jeffries et al. [9])
MAP3K14	Upregulation	Gill *O. nerka* & *O. gorbuscha* (Jeffries et al. [9])
PARK7	Upregulation	Gill *O. nerka* (Jeffries et al. [9, 30]), *O. gorbuscha* (Jeffries et al. [9])
COX6B1	Upregulation	Gill *O. nerka* (Jeffries et al. [9, 30]), *O. gorbuscha* (Jeffries et al. [9]), *O. mykiss* (Garvin et al. [101])
EIF4A2	Upregulation	Gill *O. nerka* & *O. gorbuscha* (Jeffries et al. [9]), *G. mirabilis* (Buckley et al. [64])
EIF4ENIF1	Upregulation	Gill *O. nerka* & *O. gorbuscha* (Jeffries et al. [9]) Kidney *O. mykiss* (Verleih et al. [45])
TUBA1A	Upregulation	Muscle *L. calcarifer* (Newton et al. [99])
NEK4	Upregulation	Gill *G. mirabilis* (Buckley et al. [64]; Logan & Somero, [59, 62]) Liver *F. heteroclitus* (Picard & Schulte, [102])
UBE2Q2	Upregulation	Gill *O. tshawytscha* (Tomalty et al. [17]) Kidney *O. mykiss* (Verleih et al. [45]) Liver *M. duboulayi* (Smith et al. [98])
FKBP10	Downregulation	Gill *O. nerka*, *O. gorbuscha* (Jeffries et al. [9]), *G. mirabilis (*Logan & Somero, [62]), *I. punctatus* (Liu et al. [13])
CIRBP	Downregulation	Gill *O. nerka* (Jeffries et al. [9, 30]), *O. gorbuscha* (Jeffries et al. [9]), *O. mykiss* (Rebl et al. -6]) Kidney *O. mykiss* (Verleih et al. [45])
SFRS2 / SFRS9	Downregulation	Gill *O. nerka* (Jeffries et al. [9, 30]), *O. gorbuscha* (Jeffries et al. [9])
EEF2	Downregulation	Gill *O. nerka* (Jeffries et al. [9, 30]), *O. gorbuscha* (Jeffries et al. [9]), *G. mirabilis (*Logan & Somero, [62]) Kidney *O. mykiss* (Verleih et al. [45])
SCFD1	Downregulation	Gill *O. nerka* & *O. gorbuscha* (Jeffries et al. [9])
MPDU1	Downregulation	Gill *O. nerka* & *O. gorbuscha* (Jeffries et al. [9])
IDH3B	Downregulation	Gill *O. nerka* & *O. gorbuscha* (Jeffries et al. [9]) Muscle *G. mirabilis* (Buckley et al. [64])
zgc:63572	Downregulation	Gill *O. nerka* & *O. gorbuscha* (Jeffries et al. [9])
KCT2	Downregulation	Gill *O. nerka* & *O. gorbuscha* (Jeffries et al. [9])
AP3S1	Downregulation	Gill *O. nerka* & *O. gorbuscha* (Jeffries et al. [9])
ZMYND11	–	Gill *O. nerka* & *O. gorbuscha* (Jeffries et al. [9])

**Table 3:** Summary of the initially selected candidate of thermal stress biomarkers for qRT-PCR development using gill tissue. Presented for each gene are the temperature stress response signatures, EST ID for the 44 K cGRASP microarray, and Atlantic salmon gene ID. The symbol ‘x’ indicates that the gene was included in the thermal response signature for the specified analysis

Gene Name	Gene Symbol	EST ID	Gene ID	Chromosome	Intersect 2007, 2008, 2009 (CS0101i)	Jeffries et al. [9] (ES0013)	2007 Sockeye (EX0102a)	2008 Sockeye (EX0103a)	2009 Pink (EX0101a)	Jeffries et al. [30] publication: (16 K)	Quinn et al. [32] publication Table S1 (32 K)	Anttila et al. [12] publication Table S1 (16 K)	Quinn et al. [33] publication Table S2 (32 K)	Lewis et al. [34] (24 h)	Lewis et al. [34] (4 h)
**Protein folding** and rescue															
Serpin H1 precursor	SERPINH1	C044R036	106,581,278	20	x	x	x	x	x						
		C265R019			x	x	x	x	x						
		C205R085			x	x	x	x	x						
		C101R077			x										
		CA063723								x	x				
		C009R155	106,613,072	9	x	x	x	x	x						
		C236R132			x	x	x	x	x						
Heat shock protein HSP 90-alpha	HSP90AA1	C020R155	106,608,136	6	x	x	x		x						
		DY732008									x				
Heat shock protein 90 kDa alpha (cytosolic) class B member 1	HSP90AB1	C101R112	100,136,360	15	x										
		CB493619								x	x	x	x		
		CB501628								x	x	x	x		
		CA767842									x	x			
		CB502806									x				
Heat shock protein HSP 90-alpha. 1	HSP90AA1	C198R091	106,571,588	15	x			x							
		CB498021									x	x	x		x
Heat shock protein HSP 90-alpha	HSP90AA1	C182R027	100,380,418	15	x	x	x	x							
		C015R121			x		x	x							
		CA062155									x	x	x		
Heat shock cognate 70 kDa protein	HSC71	C059R036	106,613,013	9			x		x						
Heat shock 70 kDa protein	HSP70	C035R126	106,607,232	6			x	x							
		C185R016					x	x							
		C229R092					x	x							
		C249R043					x	x							
FK506-binding protein 10 precursor	FKBP10	C171R003	106,601,011	3	x	x	x								
		C067R011	106,607,277	6	x	x	x	x							
		C165R115			x	x	x								
		C124R099			x	x	x								
		C154R080			x	x									
		C070R119	106,579,165	19	x										
		C014R004			x										
Protein disulfide-isomerase A4 precursor	PDIA4	C263R094	106,578,385	19	x										
**Oxidative stress/ion binding/signal transduction**															
Selenoprotein W	SEPW1	C035R144	106,563,412	11	x	x	x	x							
		C258R108			x	x	x	x							
		C052R159			x	x	x	x							
		C155R164			x	x		x							
		C142R109			x			x							
Mitogen-activated protein kinase kinase kinase 14	MAP3K14	C014R149	100,380,862	3	x	x		x							
		C060R057			x			x							
Parkinson disease (autosomal recessive, early onset) 7 (Protein DJ-1)	PARK7	C246R153	100,136,349	22	x	x									
**Metabolic processes**															
Cytochrome C oxidase	COX6B1	C193R155	106,579,249	19	x										
		C074R104				x									
		C042R086	106,579,245	19		x									
		C149R170				x									
Mannose-P-dolichol utilization defect 1 protein	MPDU1	C007R028	106,608,952	7	x	x	x	x							
Isocitrate dehydrogenase 3 (NAD+) beta	IDH3B	C125R081	106,565,197	12	x	x	x								
**Transcription/translation/protein transport**															
Eukaryotic translation initiation factor 4A, isoform 2	EIF4A2	C096R117	106,568,752	14	x	x	x		x						
		C092R059			x			x							
Eukaryotic translation initiation factor 4E nuclear import factor 1	EIF4ENIF1	C240R154	100,196,839	12	x	x	x	x							
Splicing factor, arginine/serine-rich 9	SFRS9	C025R120	100,195,385	1	x	x	x	x	x						
		C074R026			x	x	x	x	x						
Splicing factor, arginine/serine-rich 2	SFRS2	C078R152	106,601,525	3	x	x	x	x	x						
		C187R068			x	x	x		x						
		C254R016			x	x	x		x						
		C036R063			x	x	x	x							
		C084R083			x	x	x								
		C174R079			x		x								
		CA064151										x			
		CB493433									x				
		EG927609									x				
Eukaryotic translation elongation factor 2	EEF2	C262R107	106,569,524	14	x			x							
		CB498321								x	x				
		C102R083	106,600,009	3	x	x	x	x							
Cold-inducible RNA-binding protein B	CIRBP	C026R122	106,613,740	10	x				x						
		CA041959										x			
		C251R142	106,613,741	10	x		x								
		CA048095								x		x			
		CB499204										x			
		C107R130	106,573,600	16	x	x	x	x	x						
		C192R015	106,573,597	16	x										
Sec1 family domain-containing protein 1	SCFD1	C205R010	100,380,743	1	x	x	x	x							
**Growth regulation**															
Tubulin alpha-1A chain	TUBA1A	C036R054	100,196,438	16	x										
		C075R011													
		C062R114	106,573,955	16	x										
Tubulin alpha chain, testis-specific	TUBA1A	C145R015	106,562,989	11	x		x								
**Cell cycle and apoptosis**															
Serine/threonine-protein kinase Nek4	NEK4	C163R137	106,565,859	12	x			x							
**DNA repair and cell structure**															
Keratinocytes-associated transmembrane protein 2 precursor	KCT2	C102R089	100,194,831	13	x	x									
Ubiquitin-conjugating enzyme E2 Q2	UBE2Q2	C182R024	106,587,229	26	x	x		x							
		C089R055			x			x							
**Transmembrane transport**															
Transmembrane protein 185	zgc:63572	C160R143	106,563,673	11	x	x	x								
**Protein transporter activity**															
AP-3 complex subunit sigma-1	AP3S1	C061R023	106,585,846	24	x	x	x								
**Co-repressor of transcription**															
Zinc finger MYND domain-containing protein 11	ZMYND11	C190R138	100,380,823	3	x		x	x	x						

Among the upregulated genes, 25 of 82 selected EST identifiers were mapped to genes involved in protein folding and rescue in response to thermal stress. Seven EST identifiers from this functional group were mapped to two SERPINH1 genes (chromosomes 9 and 20). SERPINH1 was identified as a thermal stress responsive gene in 11 different studies on 8 fish species and in gill, liver, muscle and kidney tissues. Moreover, 12 EST identifiers were mapped to four HSP90 genes (chromosomes 6, and 15). HSP90 genes were reported in at least 21 publications as upregulated in response to thermal stress in 15 different fish species in seven different tissues. Five other EST IDs related to genes HSP70 and HSC71 (chromosomes 3, 6, 9, and 20) were also upregulated under thermal stress in 17 studies in 13 fish species. Finally, one EST ID mapped to protein disulfide-isomerase A4 precursor (PDIA4) on chromosome 19 was upregulated in five different fish species in response to thermal stress (Tables 2 and 3).

Eight EST identifiers were mapped to three genes involved in oxidative stress/ion binding/signal transduction, including five EST identifiers mapped to selenoprotein W (SEPW1) on chromosome 11, two to mitogen-activated protein kinase kinase kinase 14 (MAP3K14) (ch3), and one to Parkinson disease (autosomal recessive, early onset) 7 (Protein DJ-1) (PARK7) located on chromosome 22. SEPW1 is known to be upregulated in response to thermal stress in three fish species, including two salmonids. PARK7 was upregulated in two species of Pacific salmon. PARK7 was upregulated in response to thermal stress in both sockeye and pink salmon. Four EST identifiers involved in metabolic processes mapped to 2 Cytochrome C oxidase (COX6B1) genes located on chromosome 12, and were upregulated in 3 salmonid species in response to thermal stress.

In the functional group of transcription/translation/protein transport, two EST identifiers were mapped to eukaryotic translation initiation factor 4A, isoform 2 (EIF4A2) on chromosome 14, and one feature to eukaryotic translation initiation factor 4E nuclear import factor 1 (EIF4ENIF1) on chromosome 12. These genes were reportedly upregulated in 4 different fish species in response to thermal stress. Four EST identifiers involved in growth regulation were mapped to two Tubulin alpha-1A chain (TUBA1A) and tubulin alpha chain, testis-specific (Tuba1) located on chromosome 16 and 11. One EST identifiers involved in cell cycle and apoptosis was mapped to serine/threonine-protein kinase Nek4 (NEK4) (ch12). NEK4 was upregulated in 4 studies in two fish species in response to thermal stress. Finally, two EST identifiers involved in DNA repair and cell structure were mapped to ubiquitin-conjugating enzyme E2 Q2 (UBE2Q2), shown to be upregulated in 3 different fish species (Tables 2 and 3).

For the downregulated genes, 7 EST identifiers were mapped to 3 FK506-binding protein 10 precursor (FKBP10) genes located on chromosomes 3, 6 and 19. FKBP10 is involved in protein folding and rescue in response to thermal stress. The downregulation of FKBP10 has been reported for 4 different fish species when thermal stress occurred. Seventeen out of 75 EST identifiers were mapped to genes involved in transcription/translation/protein transport functional groups. Seven EST identifiers annotated to 3 cold-inducible RNA-binding protein B (CIRBP) genes on chromosomes 10 and 16. In five different studies on salmonid fishes, CIRBP was downregulated in response to thermal stress. Eleven EST identifiers mapped to splicing factor, arginine/serine-rich 9 (SFRS 9) and splicing factor, arginine/serine-rich 2 (SFRS2) on chromosomes 1 and 3, respectively. These genes were downregulated in both sockeye salmon and pink salmon in response to thermal stress. Three EST identifiers mapped to eukaryotic translation elongation factor 2 (EEF2) gene on chromosomes 14. In 4 different species EEF2 was downregulated in response to thermal stress. For this functional group, one EST identifier was mapped to sec1 family domain-containing protein 1 (SCFD1) on chromosome 1. The two EST identifiers involved in metabolic process were mapped to mannose-P-dolichol utilization defect 1 protein (MPDU1) on chromosome 7 and isocitrate dehydrogenase 3 (NAD+) beta (IDH3B) on chromosome 12. These two genes were downregulated in sockeye and pink salmon (Tables 2 and 3).

The remaining 4 selected downregulated EST identifiers mapped to genes involved in transmembrane transport, i.e. transmembrane protein 185 (zgc:63572), DNA repair and cell structure -- keratinocytes-associated transmembrane protein 2 precursor (KCT2), protein transporter activity -- AP-3 complex subunit sigma-1 (AP3S1), co-repressor of transcription -- zinc finger MYND domain-containing protein 11 (ZMYND11). These 4 genes were downregulated in sockeye and pink salmon (Tables 2 and 3).

Comparing the chronic and acute datasets in Table 3 shows that none of the identifiers from chronic studies were in the list of the Lewis et al. [34] dataset except for CB498021 that mapped to HSP90AA1. In addition, only four identifiers mapped to HSP90AA1 (Ch15) and HSP90AB1 (ch15) were in common between the chronic data sets and the Quinn et al. [33] acute dataset, while 11 identifiers mapped to SERPINH1 (ch20), HSP90AA1 (ch6), HSP90AA1 (ch15), HSP90AB1 (ch15), SFRS2, and EEF2 (ch14) (Table 3).

Given the overlap of selected candidate thermal stress biomarkers in different tissues, nine EST identifiers from the Anttila et al. [12] dataset on sockeye heart muscle mapped to HSP90a (ch15), HSP90ab1 (ch15), SFRF2, and CIRBP (Table 3).

### Testing the candidate thermal stress biomarkers

The 82 selected EST identifiers mapped to 39 unique gene IDs, related to 24 proteins. Some of the 39 gene IDs were gene paralogs (e.g. SERPINH1, HSP90, etc.) that may be acting differently. In total, 46 TaqMan assays were designed for the 39 genes. Two assays were designed for some of the gene IDs for further efficiency testing and thermal stress biomarker validation (Table 4). The 46 designed assays were validated on the BioMark microfluidics qRT-PCR Platform, 43 of which worked well across all six salmonid species with efficiencies between 0.65 and 1.5. Assays with efficiencies between 0.8 and 1.1 were passed. Thirty-two unique biomarkers that showed the best PCR efficiencies across all tested species were selected (Table 4).

**Table 4:** TaqMan assays and PCR efficiencies of selected genes outlined in Table 3 across 6 salmonid species

Symbol	Chromosome	Primers and TaqMan Probes	PCR Efficiency					
			*O. tshawytscha* (Chinook)	*O. kisutch* (Coho)	*O. keta* (Chum)	*O. gorbuscha* (Pink)	*O. nerka* (Sockeye)	*S. salar* (Atlantic)
SERPINH1	20	F-ACTATGACCACTCGAAGATCAACCT R-CCCATTCGTTGATGGAGTTCA P-AGGGACAAGAGGAGC	0.75	0.90	0.87	0.79	0.89	0.84
SERPINH1	9	F- GAGGTCAGCGACCCAAAGAC R- GCCGTAGAGGCGGTTACTGAT P- CGGAACGTCACATGGA	0.84	0.97	0.96	0.95	0.92	1.08
HSP90AA1	6	F- TTGGATGACCCTCAGACACACT R- CGTCAATACCCAGGCCTAGCT P- CCGAATCTACCGGATGAT	0.91	0.88	0.98	0.98	0.87	0.87
HSP90AB1	15	F- GACACGGTGTTGGGTTGGTT R- TTGCAGTCAACTCTCCATGCA P- TCATGTGCAACATAACAT	0.86	0.85	0.78	0.88	0.83	0.77
HSP90AA1	15	F- ATGACCCTCAGACACACTCCAA R- CCTCATCAATACCCAGTCCTAGCT P- CGCATCTACAGAATGA	0.82	0.70	0.68	1.00	0.85	0.84
HSC71	9	F- CTTAGGGACGCCAAGATGGA R- GAGCCTCCCACCAGGACAA P- AAGCCCAGGTCCACGAC	0.90	0.86	1.33	0.94	0.86	0.82
HSP70	6	F- TCAACGATCAGGTCGTGCAA R- CGTCGCTGACCACCTTGAA P- CCGACATGAAGCACTGG	0.88	1.02	0.81	0.89	0.82	0.65
PDIA4	19	F- TGAGGTGCAGGACTTTTTTAAGAA R- TCGTTGCTCTGTTTCCTGTGA P- ACATCCTGCCACTGGT	0.90	0.88	0.81	0.90	0.86	0.93
SEPW1	11	F- TGAGGATGAATTCCCAGGTGAT R- AAACCACCCAGAGGTTGAAGGT P- TTGAGATTACTGGTGAAAGC	0.84	0.83	0.73	0.91	0.84	0.87
MAP3K14	3	F- GCTCCCTGGGTTCATGGAT R- GCCTCCCTTCAGCAGAGACA P- CCAGCAATAGCTTATG	0.93	0.78	0.78	1.09	0.85	1.03
PARK7	22	F- ACTGCAAGCAGCATGATCAACT R- TTGGCCTGTGTATCATAATGAACA P- CCCCACCTACTCAGC	0.94	0.85	0.81	0.97	0.91	–
COX6B1	19	F- GCCCCGTGTGACTGGTATAAG R- TCGTCCCATTTCTGGATCCA P- TCTACAAATCACTGTGCCC	1.05	0.90	0.86	0.91	0.85	0.75
EIF4A2	14	F- CAGAAACTGAGCACAAACATTCAA R- GGACATCTGCAGGCATGGTA P- TGGTCCTCCTCTCTG	0.90	0.99	0.87	0.86	0.89	0.73
EIF4ENIF1	12	F- AGCTCCATCCCAGCCTTGT R- AAATATGCCTGCCTGCATCAG P- CCAGGGCATCCAGCCA	0.75	0.86	0.74	–	0.71	1.16
TUBA1A	16	F- CTCTGCTGAGAAGGCCTACCAT R- AGCAGGCGTTGGTGATGTC P- AGCAGCTGTCTGTTGC	0.90	0.86	0.79	0.93	0.86	0.86
TUBA1A	11	F- AGACCGGAGCGGGAAAAC R- ACAACAGTTGGTTCGAGATCCA P- TGTCCCCCGTGCTGT	1.10	0.87	0.83	0.86	0.87	1.48
NEK4	12	F- TGCGGCAGCTAAAATTCTTTG R-AATGCATTTTTCATTAGCTGATCCT P-AGACGTTTCTTTCAAGGGT	0.78	0.92	0.67	0.84	0.85	1.17
UBE2Q2	26	F- GGCAGGACCACTTGAACGTAA R- AGGCCTGCACTGAACCAGAT P- TGCTCATTCGGGTGCG	0.92	1.13	0.80	1.08	0.87	0.75
FKBP10	3& 6	F- ACTATGAGAATGCCCCCATCAC R- CTCGTCCAGACCCTCAATCAC P- CCTGGGAGCCAACAA	0.89	0.88	0.86	1.05	0.85	0.66
FKBP10	19	F- CCTGAAGAGATCATTGCTGACATG R- GACGATGACCCCATCCTTGT P- TCAGGAACCAGGACCG	0.97	0.82	0.84	0.76	0.89	0.80
CIRBP	16	F- GGGATGGTGGAGACCTTCTCT R- CAGAACCCACAGCGATCCTAA P- TTCTCTAGTCCACTGGGCT	2.13	0.89	1.83	–	1.56	–
CIRBP	10	F- TGATTGACTGTTTTGCCAACTGA R- TCAGACCTTTGTGTGCATTTACCT P- ATGGTGATGAGCCTGAAT	1.06	0.81	0.83	0.87	0.84	0.85
SFRS9	1	F- ACATTCGTGTCCACGGAGAAC R- GGACCCTCTGCTTTTGTAAGGA P- TGCCAGTTATGGTCGCT	0.85	0.98	0.95	1.27	1.04	0.91
SFRS2	3	F- TCCAGATGGCCCGTTACG R- CACCACCGCCTCCATGAT P- TCCCCCAGATTCT	0.87	0.85	0.82	0.90	0.85	0.81
EEF2	14	F- AGGTCACAGCCGCCCTTAG R- ACACAGTCTCTGTCTGCACACACA P- CGACTGCGTCTCAGGT	0.88	0.87	0.82	0.89	0.84	0.89
SCFD1	1	F- GACAAGAAGCTGAGGGAGAACCT R- GCCGGCCCCCATATTG P- ACAGCCTGTTCACTGGA	0.84	0.88	0.89	0.99	0.88	0.73
MPDU1	7	F- TGCTTGACCCCTTGATTATAGCTA R- GACCATAATCTAGAATGAAAACGCATT P- CTTCCTGGTTGTGTTCTG	0.93	0.78	0.78	1.09	0.85	1.03
IDH3B	12	F- AGAAATCTCTACCACAGCACTGTATCA R- GGCACGACTCAGGACTGTGA P- TGGATATCTGGCCTGTCAT	0.85	0.85	0.83	0.88	0.85	0.78
zgc:63572	11	F- GGCTATCCCTGGTGACTCTGAT R- ACCAGTGGTTGCCTCCTTTCT P- CCACGACCTTCGGC	0.92	0.87	0.72	0.94	0.87	0.87
KCT2	13	F- TGTTCTCACCATGTTTAAGCCTTACT R- TGCAATCAGCACAAACACTTAAGA P- CTTCTACACTTATTGTTTTGC	0.85	0.90	–	–	0.83	0.86
AP3S1	24	F- TGGCATGTTGTTAGCCTGCTA R -TCTACCTGGGAACCGACTCTAATC P- CCGTAATTTGAGCTAGATTG	0.88	0.88	1.00	1.22	1.03	1.00
ZMYND11	3	F- TCGCCCCACTGTCACTCA R- GATTCGGTCCACAAAGTGTTCA P- ACACCCCTACCTGCCTT	0.87	0.82	0.75	1.09	0.79	0.68

### Expression of thermal stress biomarkers in 2007 sockeye salmon

**Fig. 2:**
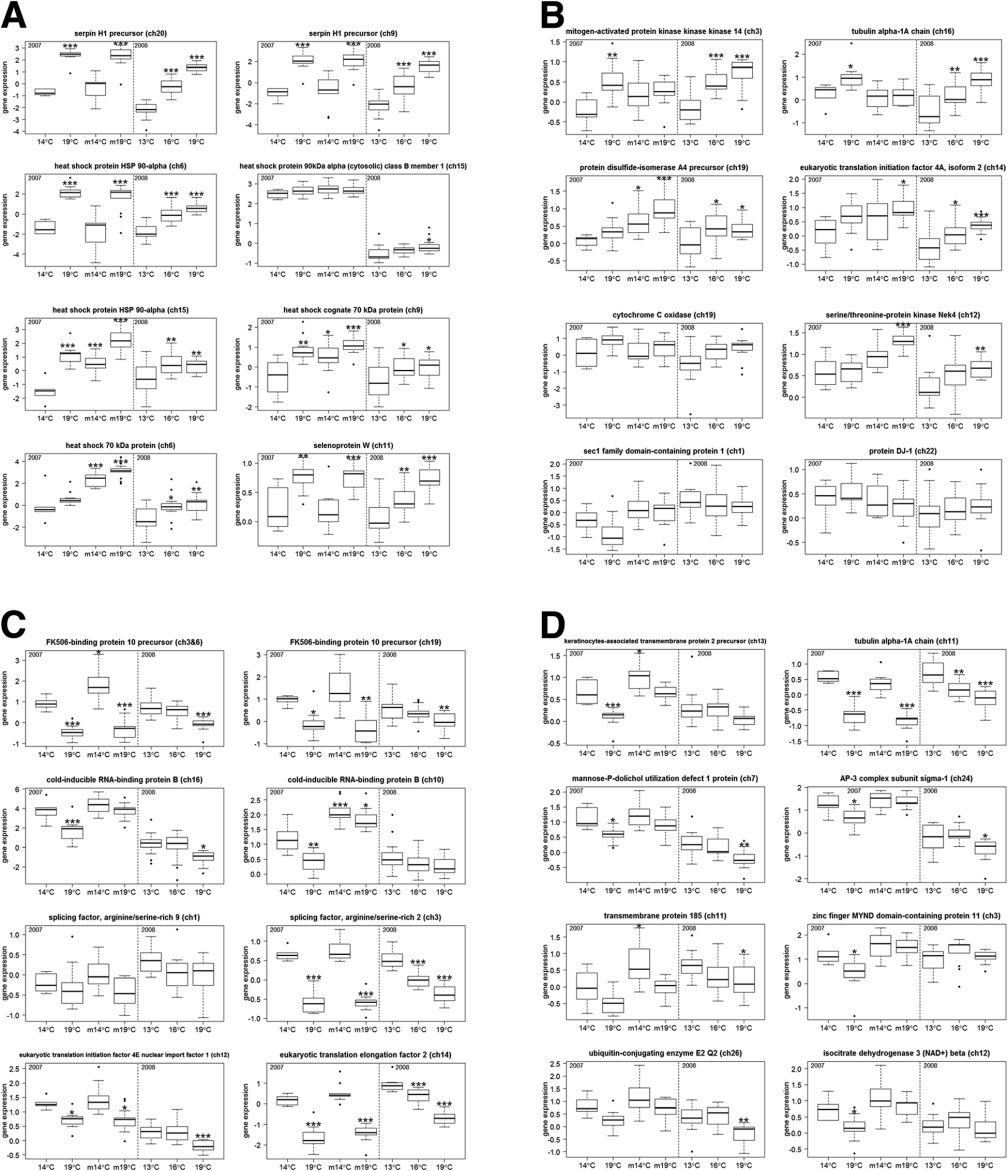
Gene expression box plots of thermal stress biomarkers for adult sockeye salmon held at cool and warm temperature conducted in 2007 and 2008. m = moribund. *P < 0.05; **P < 0.01; ***P < 0.001

The 2007 thermal challenge study used for biomarker validation included fish that survived to the end of each study and fish that became moribund before or at the end of the study, and these groups showed a somewhat different response to thermal biomarkers. Among those biomarkers showing consistent responses to thermal stress among survivors and moribund sockeye salmon, six biomarkers showed significantly higher expression in fish held at 19 °C compared to fish held at 14 °C, including SERPINH1 (ch20), SERPINH1 (ch9), HSP90AA1 (ch6), HSP90AA1 (ch15), HSC71 (ch9) and SEPW1 (ch11). Four biomarkers showed significantly lower expression in fish held at 19 °C compared to 14 °C for both survivors and moribund sockeye salmon, including FKBP10 (ch3&6), SFRS2 (ch3), EEF2 (ch14), and TUBA1A (ch11). Four biomarkers only separated thermal groups in surviving fish. One of these, MAP3K14 (ch3), showed significantly higher expression in survivor fish held at 19 °C compared to 14 °C, while three, CIRBP (ch16), CIRBP (ch10) and KCT2 (ch13), showed significantly lower expression in survivor fish held at 19 °C compared to 14 °C. Four biomarkers showed separation between thermal treatments only in moribund fish. Three were upregulated, HSP70 (ch6), PDIA4 (ch19) and NEK4 (ch12), and one, FKBP10 (ch19), was downregulated in moribund fish held at 19 °C compared to 14 °C. No significant differences were observed in 14 candidate thermal stress biomarkers including HSP90AB1 (ch15), TUBA1A (ch16), EIF4A2 (ch14), COX6B1 (ch19), MPDU1 (ch7), SFRS9 (ch3), PARK7 (ch22), EIF4ENIF1 (ch12), AP3S1 (ch24), UBE2Q2 (ch26), IDH3B (ch12), ZMYND11 (ch3), SCFD1 (ch1), and zgc:63572 (ch11) (Fig. 2). The significance cut-off was *P* < 0.01 for these analyses.

### Expression of thermal stress biomarkers for 2008 sockeye salmon

The thermal challenge study in 2008 only included fish that survived to the end of the challenge. Eleven biomarkers showed significantly higher expression in fish held at 19 °C compared to fish held at 13 °C, including SERPINH1 (ch20), SERPINH1 (ch9), HSP90AA1 (ch6), HSP90AA1 (ch15), HSP70 (ch6), EIF4A2 (ch14), COX6B1 (ch19), SEPW1 (ch11), MAP3K14 (ch3), TUBA1A (ch16) and NEK4 (ch12). In contrast, 8 biomarkers showed significantly lower expression in fish held at 19 °C compared to 13 °C including FKBP10 (ch3&6), FKBP10 (ch19), SFRS2 (ch3), EIF4ENIF1 (ch12), EEF2 (ch14), MPDU1 (ch7) UBE2Q2 (ch26) and TUBA1A (ch11). No significant differences were observed in 13 candidate thermal stress biomarkers including HSP90AB1 (ch15), HSP70 (ch6), CIRBP (ch16), CIRBP (ch10), SFRS9 (ch3), SCFD1 (ch1), IDH3B (ch12), PARK7 (ch22), KCT2 (ch13), ZMYND11 (ch3), AP3S1 (ch24), zgc:63572 (ch11) and PDIA4 (ch19) between treatments (Fig. 2). All significant differences were considered as *P* < 0.01.

### Expression of thermal stress biomarkers for Chinook salmon

**Fig. 3:**
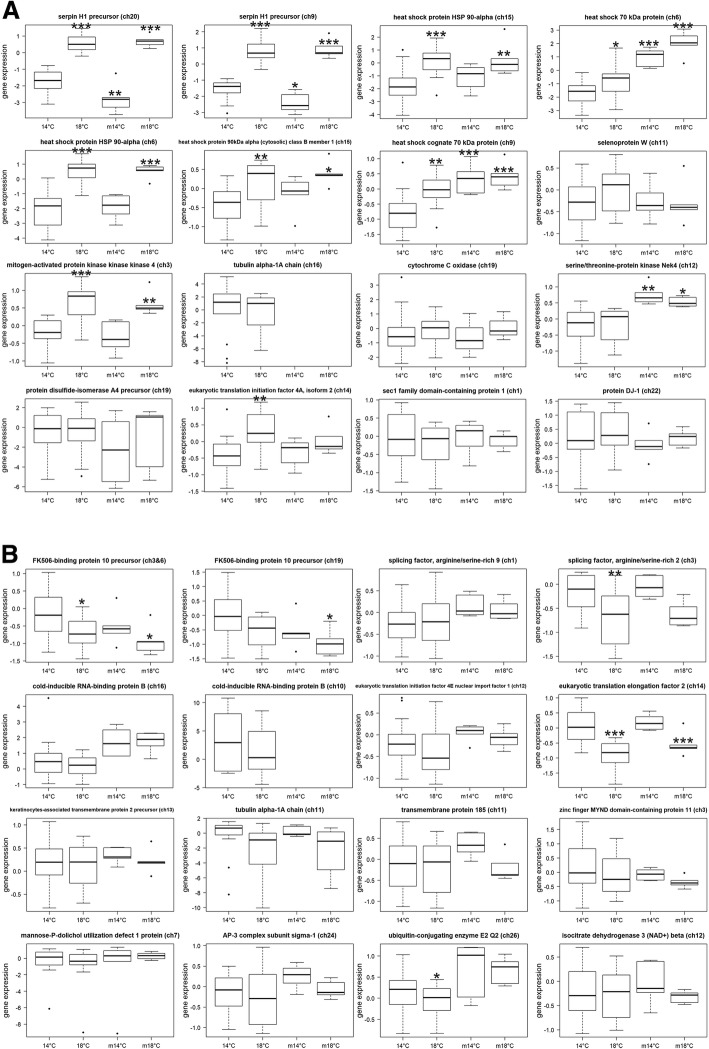
Gene expression box plots of thermal stress biomarkers for juvenile Chinook salmon held at cool and warm temperature conducted in 2017 and 2018. m = moribund. *P < 0.05; **P < 0.01; ***P < 0.001

Similar to the 2007 thermal challenge study on adult sockeye salmon, gill samples of juvenile Chinook salmon that survived to the end of study and fish that became moribund before or at the end of the study were used for biomarker validation (Fig. 3). Among those biomarkers showing consistent responses to thermal stress among survivors and moribund Chinook salmon, six biomarkers showed significantly higher expression in fish held at 18 °C compared to fish held at 14 °C, including SERPINH1 (ch20), SERPINH1 (ch9), HSP90AA1 (ch6), HSP90AA1 (ch15), HSC71 (ch9) and MAP3K14 (ch3). On the other hand, only EEF2 (ch14) showed significantly lower expression in fish held at 18 °C compared to 14 °C for both survivors and moribund Chinook salmon. Three biomarkers only separated thermal groups in surviving fish. Two, HSP90AB1 (ch15) and EIF4A2 (ch14) showed significantly higher expression in survivor fish held at 18 °C compared to 14 °C, while SFRS2 (ch3) showed significantly lower expression in survivor fish held at 18 °C compared to 14 °C. Two biomarkers including HSP70 (ch6) and NEK4 (ch12) were upregulated only in moribund fish held at 19 °C compared to 14 °C. No significant differences were observed in remaining 20 candidate thermal stress biomarkers. The significance cut-off was P < 0.01 for these analyses.

## Discussion

The rapid development of functional genomic technologies designed to explore changes in gene expression has increased our understanding of the genetic regulatory underpinnings of cellular and organismal responses to abiotic stressors [35]. Our study sought to mine the extensive functional genomic studies in fishes to identify robust thermally-responsive biomarkers that could provide the basis of a monitoring tool to characterize chronic thermal stress in wild-caught salmon. The extensive microarray literature was mined to identify a subset of 82 chronic thermal stress biomarkers differentially activated in gill tissue. TaqMan assays for 32 unique genes, including some gene paralogs, with good efficiencies across salmon species were developed. Their expression in response to temperature holding in thermally challenge sockeye and Chinook salmon was assessed. Eight genes including SERPINH1 (ch9), SERPINH1 (ch20), HSP90AA1 (ch6), HSP90AA1 (ch15), FKBP10 (ch3&6), MAP3K14, SFRS2, and EEF2 (ch14) consistently showed strong and robust chronic response to temperature stress in the discovery analysis and both sockeye and Chinook salmon validation studies, and can be considered as biomarkers to diagnose exposure to thermal stress. Excluding MAP3K14, and SFRS2 that did not show differences in moribund sockeye and Chinook salmon, respectively, all the remaining six biomarkers, together with HSP70 (ch6) and NEK4 (ch12) can also be utilized as biomarkers for thermal stress in moribund fish.

Our aim was to develop a biomarker-based tool that can monitor molecular physiological signatures of chronic thermal stress in fish gill tissue. Fish gills are a multifunctional organ which directly contacts the external environment and can be sampled non-destructively [36, 37]; these features make gill an ideal target for monitoring responses of fishes to environmental stress, even in applications on fishes of conservation concern. Moreover, non-lethal sampling means that molecular assessments can be performed in conjunction with tagging (e.g. Miller et al. [29], and Cooke et al. [38]) or holding (e.g. Jeffries et al. [30]) studies, or in situations where repeat sampling is warranted (e.g. Teffer et al. [23]). We demonstrated that fish gill exhibits a robust molecular physiological response to high temperature exposure based on the number of genes showing modulated expression, the level of gene expression, and the range and pattern of observed response.

Unsurprisingly, heat shock response genes were among the most upregulated genes both from the literature and specifically in our validation study of sockeye and Chinook salmon experiencing chronic thermal stress. The enhanced presence of HSPs in response to thermal shock is a well-investigated research area in fishes. The protein family is highly conserved and present in all organisms that have been examined [39]. In response to an initial stress exposure, cells accumulate HSPs since they are crucial for the maintenance of the protein’s integrity. They protect tissues from structural damage during subsequent exposures to stressors [40]. Published studies and microarray discovery analysis showed that HSPs are responsive to both acute and chronic stress, among which SERPINH1 (serpin peptidase inhibitor, clade H, member 1, also known as HSP47), HSP90 and HSP70 were found the most frequent HSPs expressed in response to thermal stress across salmonids and other species studied. In sockeye and Chinook salmon gill, two transcripts each of molecular chaperones SERPINH1 and HSP90AA1 were among the top upregulated genes in fish held at 18–19 °C versus 13–14 °C, significant in both studies. Moreover, these genes have been previously described in Pacific salmon in response to chronic thermal stress [9, 28, 30, 41].

In our study, SERPINH1 genes mapped on chromosomes 9 and 20 were both activated at similar intensity in response to thermal stress, based on discovery analysis and BioMark gene expression analysis. SERPINH1 is a collagen-specific molecular chaperone localized in the endoplasmic reticulum (ER) [42], and is essential for the proper assembly of the triple-helical procollagen molecules [43]. SERPINH1 is expressed in all collagen-synthesizing cells, and constitutive expression levels correlate strictly with the amounts of collagen being synthesized in the corresponding cell [43, 44]. SERPINH1 is a well-established heat inducible protein and its synthesis increases strongly under heat-shock conditions, being regulated by a heat shock element in its promoter region; constitutive and tissue-specific expression of SERPINH1 correlates with that of collagen and is regulated via enhancer elements located in the promoter and intron regions [42]. SERPINH1 has been reported to be strongly induced following exposure to chronic thermal stress in multiple fish species, including *O. nerka* [9, 30], *O. gorbuscha* [9], *O. mykiss* [6, 7, 45], *O. tshawytscha* [17], *Hypomesus transpacificus* [16], *Gadus morhua* [46], *Pomacentrus moluccensis* [47], and *Puntius sophore* [48]. Our thorough literature review showed that SERPINH1 has not been recognised as an upregulated gene in response to any other stressors in fish, although in mammals it is also associated with fibrosis in response to parasite infections [49]. Therefore, the upregulation of SERPINH1 genes could be considered as a specific biomarker for thermal stress in fish.

Many EST identifiers mapping to HSP90AA1 and HSP90AB1 were resolved in our discovery analysis, but only HSP90AA1 (ch 6&15) validated by TaqMan assays. Transcripts of HSP90AA1 showed significant upregulation in the thermal stress group compared with controls. HSP90AA1 is an abundant, well-conserved cytosolic protein that accounts for 1–2% of all cellular proteins in most cells under basal, non-stress conditions with levels increasing in response to heat stress and other proteotoxic insults [50]. HSP90AA1 plays a key role in the response of cells to stress and is thought to be important in buffering cells against the effects of mutation [51–55]. However, HSP90AA1 is upregulated in response to multiple different stressors, hence may not, on its own, be a reliable predictor of heat stress. Increased expression of *HSPs* in fishes is well documented for heat shock, anoxia or hypoxia and microbial infection [12, 32, 33, 56–59].

Seven transcripts mapped to FKBP10 were significantly downregulated in both live and moribund sockeye salmon in higher temperature. FKBP10 was also significantly downregulated in both live and moribund juvenile chinook salmon when the significance cut-off was *P* < 0.05. FKBP10 encodes FKBP65, another type I procollagen chaperone resident in the ER. Duran et al. [60] showed that FKBP65 acts at a similar stage during type I procollagen maturation as SERPINH1. An interaction between FKBP65 and SERPINH1 confers mutual molecular stability and also allows for a synergistic effect during collagen folding [61]. It has been known that the chaperon function of FKBP65 in the ER may actually be predominantly fulfilled by SERPINH1 [61]. Therefore, a remarkable of increase of SERPINH1 expression in fish under thermal stress might explain downregulation of FKBP10 gene. We suggest that differential expression of the FKBP10 genes could be an indicator of thermal stress response across fish species. Differential expression of FKBP10 was consistent with responses documented in *G. mirabilis* [62], *I. punctatus* [13] and *Acipenser* sp. [63] in response to high temperature. Differential expression of FKBP10 could be specific for thermal stress, hence could be a strong contributor towards a biomarker panel to predict chronic thermal stress in fish. The chaperone activity of FKBP65 is comparable to that of PDIA4. PDIA4, a chaperone involved in folding secreted proteins in the ER, was moderately upregulated in both 2008 survival and 2007 moribund fish in 19 °C compared to 13–14 °C. Although, the upregulation of protein disulfide isomerase in response to elevated temperatures is well known in *O. nerka* [30], *O. tshawytscha* [17], *G. mirabilis* [62, 64], *Trematomus bernacchii* [65] and *I. punctatus* [13], PDIA4 induction has also been reported when fish are exposed to other environmental stressors. For example, differential expression of PDIA4 was reported in response to salinity increase in Nile Tilapia, *Oreochromis niloticus* [66], exposure to Tris (1,3-dichloro-2-propyl) phosphate (TDCIPP) [67], and methylmercury [68] in zebrafish (*D. rerio*). Therefore, this gene might not be a specific biomarker for thermal stress in fish, but may work as part of a co-expressed panel.

SEPW1 was also significantly upregulated at 19 °C compared to 13–14 °C in both survivors and moribund sockeye salmon in both trials. SEPW is a low molecular weight and selenocysteine containing protein with redox activity involved in the antioxidant response [69]. It can serve as an antioxidant, responds to stress, is involved in cell immunity, is specific target for methylmercury, and has thioredoxin-like function [70]. Oxidative stress is common in organisms during periods of extreme environmental challenges, including temperature stress [71]. An increase in metabolism at higher temperatures may create more Reactive Oxygen Species (ROS) and therefore oxidative stress [35].

MAP3K14, which specifically regulates the stress responsive nuclear factor kappa-B (NF-kB) pathway through protein phosphorylation, was also significantly upregulated at 18–19 °C compared to 13–14 °C in both 2007–2008 sockeye and 2017–2018 Chinook trials. NF-κB is essential for cell survival after heat shock by activating autophagy, a mechanism that probably helps the cell to cope with hyperthermic stress through clearance of damaged proteins [72]. It is also known that NF-κB signaling plays a crucial role in preventing heat stress-induced early apoptosis [73]. Therefore, MAP3K14 levels may indicate exposure to high water temperatures in fish.

Two genes involved in metabolic processes were significantly differentiated in 2008 samples in response to higher temperature, COX6B1 and MPDU1, consistent with the results of discovery analysis. COX6B1, a non-transmembrane subunit of COX that faces the mitochondrial intermembrane space, stabilizes COX dimerization along with COX6A [74]. The upregulation of COX6B1 could be related to thermal stress induced oxidative stress. Chronically elevated temperatures result in increased cardiac output as well increases in mitochondria metabolic rates and whole animal oxygen consumption. Such increases in metabolic rates and oxygen consumption can lead to increased production of ROS by mitochondria [75]. In addition, stress can result in reduced cellular oxygen levels triggering enhanced mitochondrial ROS production. These factors also predict an increased need for antioxidant defenses [76]. Exposure to acute increases in temperature results in transient oxidative stress and changes in antioxidant enzyme activities, as demonstrated in several fishes [77–79].

Exposure to sufficiently high temperatures can inhibit general protein biosynthesis, concomitant with the preferential production of specific stress response proteins [64]. Heat stress is known to repress transcription and protein synthesis, probably reflecting the suppression of noncritical activities during stress [80]. These responses, however, can vary among genes [47]. Our results showed that genes involved in protein biosynthesis, including EEF2 and SFRS2 were significantly downregulated in fish held in higher temperature, consistent with reduced protein biosynthesis during thermal stress. Previous studies across taxa have shown that exposure to chronic elevated water temperatures can decrease protein biosynthesis and this response may be more pronounced in fish with limited energy availability, as would be the case with adult Pacific salmon that rely solely on endogenous energy stores to fuel freshwater migration [9, 30]. A downregulation of nonessential protein biosynthesis may be a strategy to conserve finite energy stores during a temperature-induced increase in metabolic rate [9], which may ultimately allow the organism to cope with prolonged exposure to thermal stress [47].

Our results showed that three EST identifiers mapped to Tubulin alpha-1A chain gene showed differential expression in higher temperature in both 2007 and 2008 sockeye salmon. Tubulin is the major constituent of microtubules. The upregulation of Tubulin alpha contribute to stabilization of the cytoskeleton in response to changing temperatures. The upregulation of this gene has also reported in killifish *Austrofundulus limnaeus* in response to constant high temperature but not cycling temperatures [81] The other Tubulin alpha gene, Tubulin alpha chain, testis-specific on chromosome 11 also showed differentially expression in response to thermal stress in sockeye salmon, but the transcripts of this gene were downregulated. Tubulin alpha-1A chain therefore could be considered as a reliable biomarker for thermal stress in fish.

The results of the qRT-PCR analysis of thermal stress biomarkers successfully validate the discovery analysis. In the discovery analysis, several EST identifiers coding for SERPINH1 (ch9), SERPINH1 (ch20), HSP90AA1 (ch6), HSP90AA1 (ch15), FKBP10 (ch3&6), MAP3K14, SFRS2, and EEF2 (ch14) genes showed strong and robust chronic temperature stress response biomarkers. Consistently, all of the stated genes were among the most differentially activated genes in gill tissue of Pacific salmon in response to elevated temperatures in sockeye salmon for both 2007 and 2008 and Chinook salmon challenge experiments. Among the validated thermal stress biomarkers, genes involved in chaperoning and protein rescue, protein biosynthesis, and oxidative stress were induced by chronic thermal stress in sockeye salmon and when expressed in concert, could be considered as reliable candidate biomarkers for thermal stress. A similar biomarker approach has been developed and validated for biomarkers predictive of viral disease development (VDD) across multiple species of RNA viruses [22]. Eight of the discovered biomarkers, including HSP90AB1, SFRS9, SCFD1, PARK7, IDH3B, AP3S1, zgc:63572, and ZMYND11 did not validate as robust thermal responsive biomarkers (*P* > 0.01), however, most of these genes showed significant up- or downregulation at *P* < 0.05 via qRT-PCR in Sockeye salmon. Moreover, although, the EST identifiers mapped to these genes showed consistent fold change direction across the 3 years in discovery analysis, many of them showed lower fold changes in 2007 and 2008 sockeye salmon than 2009 pink salmon. For example, several EST identifiers coding for HSP90AB1, SCFD1, ZMYND11, showed lower fold changes in 2007 and 2008 sockeye salmon than corresponding fold changes for pink salmon (2009). Therefore, it is likely that the biomarkers that did not validate well across species were largely derived from pink salmon microarray studies, a species not examined in our validation analysis.

Our reason for using data from different species and tissues in the discovery analysis was to help with the identification of general temperature response markers across tissues. Four responsive genes to thermal stress in the muscle heart tissue of sockeye salmon [12] showed overlap with the selected candidate thermal stress biomarkers discovered in gill tissue, indicating that there is some similarity between different tissues and cells in modulation of gene expression in response to thermal stress. Moreover, our deep literature mining on published studies in multiple fishes also indicates that genes i.e. SERPINH1, HSP90, HSP70, PDIA4, EIF4ENIF1, CIRBP, EEF2 and IDH3B showed similar response to thermal stress in different tissues (see Table 2).

Additionally, our study suggests that except for HSP90 genes, the proposed seven biomarkers identify chronic climate induced temperature stress. Comparing two acute thermal stress data sets [33, 34] and the chronic datasets used in the discovery analysis showed no overlap except for EST identifiers mapped to HSP90 genes. However all the EST identifiers in common between the Quinn et al. [32] chronic dataset and other chronic datasets used in the discovery analysis mapped to SERPINH1 (ch20), HSP90AA1 (ch6), HSP90AA1 (ch15), HSP90AB1, SFRS2, and EEF2 (ch14) which were among the seven thermal stress biomarkers.

It must be taken into account that all the discussion about the function of the proteins is based on the assumption that the gene and protein expression are tightly coupled. Moderate correlations between gene expression and protein activity are also common [82, 83], possibly because of post-transcriptional and post-translational modifications [84]. However, changes in mRNA levels may be one of the first indicators of a physiological change or response [22, 85]. It is true that the expression of a single gene does not necessarily correlate with the corresponding protein expression, however up- or downregulation of a panel of genes with the same function usually delivers important information on the system.

## Conclusions

Altogether, independent qRT-PCR validation of biomarkers discovered through meta-analysis of microarray data generated a panel of eight genes involved in chaperoning and protein rescue, protein biosynthesis, and oxidative stress that were differentially activated in gill tissue of Pacific salmon in response to elevated temperatures and could serve as reliable specific thermal stress biomarkers. While individually, some of these biomarkers may also respond to other stressors or biological processes, when co-expressed in concert, these genes could provide a reliable method to specifically detect the presence of a thermal stress response in field-caught salmon. Examining suites of genes characteristic of a specific response represents a more powerful approach for assessing the effects of environmental stressors across species [18]. The next step will be to demonstrate the specificity of these biomarkers under a multi-stressor challenge, and to determine the minimum number of genes required to specifically predict a thermal stress response.

## Methods

Candidate thermal stress biomarkers for gill tissue were resolved through combination of results from two separate approaches: (1) discovery analysis based on microarray data, and (2) deep literature mining. The goal of the discovery analysis was to extract a set of robust candidate biomarker genes from microarray data depicting responses to acute and chronic temperature stress. New bioinformatic analyses were performed on cGRASP 44 K microarray datasets from the Molecular Genetics Laboratory (MGL), Pacific Biological Station, Nanaimo, BC derived from thermal challenge holding studies of adult Pacific salmon species. The discovered signatures resolved from these analyses were then compared with signatures of external, published microarray studies on chronic and acute temperature stress in salmon, as shown in Table 5. The literature mining approach compared significant genes across published studies in all fish species, and selected the genes that were listed as significant at least twice in any of the papers.

**Table 5:** Overview of temperature studies and data sets used in discovery analysis

Data sets/studies	Species	Study design	Target tissue	Signature(s) used in discovery	Data used in discovery	Platform	GEO
Jeffries et al. [30]	Sockeye salmon	16 control (14 °C) and 24 high temperature exposed (19 °C) fish for 1 week (Chronic)	Gill	✓	✓	GRASP16K (GPL2716)	GSE33586
Jeffries et al. [9]	Pink and Sockeye salmon	49 control (13–14 °C) and 49 high temperature exposed fish (19 °C) for 5–7 days (Chronic)	Gill	✓	✓	cGRASP44K (GPL11299)	GSE42558
Quinn et al. [33]	Arctic charr	6 control (6 °C) and 12 high temperature exposed (25 °C) fish for 6–8 h (Acute)	Gill	✓	✓	cGRASP32K (GPL10096)	GSE26306
Quinn et al. [32]	Arctic charr	10 control (6 °C) and 10 high temperature exposed (15–18 °C) fish for 72 h (Chronic)	Gill	✓		cGRASP32K (GPL10096)	GSE29610
Anttila et al. [12]	Sockeye salmon	4 control (12–13 °C) and 4 high temperature exposed fish (18–19 °C) for 3 days (Chronic)	Heart muscle	✓		GRASP16K (GPL3976)	GSE50054
Lewis et al. [34]	Rainbow trout	6 control (11–13 °C) and 6 high temperature exposed (25 °C) fish for 4 h and 24 h (Acute)	Red blood cells	✓		GRASP16K (GPL2716)	GSE21084

Assessment of variation across studies using different array platforms is somewhat hampered by differential gene compositions and annotation, the extensive use of synonyms to describe the same gene, and the potential impact of splice variants and gene paralogs. Hence, we started our analyses by annotation-mapping across the targeted arrays so that we can identify the common sets of genes probed. This involved mapping the probe sequence data for each array platform applied in salmon to the Atlantic salmon genome (assembly ICSASG_v2) and re-annotating the arrays to include genome location. We did this across the salmon GRASP 16 K and 32 K arrays, the cGRASP Salmonid 44 K array, and the SIQ and TRAITS arrays. This enabled not only common name usage across arrays, but in most cases also allowed us to differentiate gene paralogs and probes that comprise the same exons(s) of a given gene, thus reducing the impacts of splice variants on data reproducibility.

### Selection and preparation of data sets

The study overview in Table 5 summarizes relevant temperature-related studies, data sets and signatures that were applied to discovery analysis. Our main focus was on studies based on gill tissue of salmon species, as the tool we were looking to build was to be based on gill tissue, but signatures from Anttila et al. [12] (heart) and Lewis et al. [34] (red blood cells) were used to assess tissue overlap in comparison analyses. Bioinformatics analyses were conducted on data and signatures from six published studies to resolve biomarkers strongly associated with temperature stress. A gene signature from Rebl et al. [6] derived from a non-GRASP platform was also included, and used for a comparison against the proposed candidate gene list. Data sets were downloaded from the Gene Expression Omnibus (GEO) where available, as indicated in Table 5, and published signatures were downloaded or parsed from respective supplementary materials. Internal microarray data sets from thermal challenge studies included 21 sockeye salmon gill samples from 2007, 33 sockeye salmon gill samples from 2008, 44 pink salmon (*O. gorbuscha*) gill samples from 2009, and 40 sockeye salmon samples from Jeffries et al. [30] were used (Table 5).

### Outline of analyses

A computational pipeline with data exploration, biomarker discovery and validation functionality was used to perform the analyses. Exploration analyses were performed on all data sets using projection-based methods such as Principal Components Analysis (PCA) [86] to get an overview of the data and potential outlier samples. Unsupervised methods with feature selection such as Gene Shaving [87] or sparse PCA [88] were used for further exploration and to identify additional signatures that showed separation of samples by temperature without the use of supervised labels.

The discovery analysis were run on cGRASP44K and cGRASP32K analysis data sets, including 98 samples from the MGL 2007–2009 temperature gill sockeye (2007 and 2008) and pink (2009) adult salmon challenge studies that were used in Jeffries et al. [9]. From this analysis, we defined a signature of 49 EST identifiers commonly found in separate analyses of the low (13–14 °C) and high (19 °C) temperature data. A re-analysis of the same data using robust limma [89] instead of ANOVA (which was used in Jeffries et al. [9]) was performed to confirm published results and to provide a potentially larger signature, as the robust limma method is more lenient with outliers when determining significance. This analysis produced three discovery signatures (DS0101, DS0102 and DS0103) as indicated in Table 6. The three signatures were combined using the intersection (CS0101i) and union (CS0101u) methods, and combined signatures were compared with published signatures. Gene Shaving and sparse PCA exploration methods were applied to determine additional exploratory signatures for the 2007 sockeye (EX0102a), the 2008 sockeye (EX0103a) and the 2009 pink (EX0101a) salmon data (Table 6). Sparse PCA is similar to PCA, but it allows putting sparsity constraints on the input variables so that a pre-specified number of variables can be selected and returned by the method. For all sparse PCA discovery analysis, 100 EST identifiers were selected in each dimension.

**Table 6:** Temperature stress response discovery analysis. Discovery analysis utilized the MGL 2007-2009 Temperature data set (cGRASP44K) from Jeffries et al. [9], and publicly available data from the Quinn et al. [32] study on chronic, sub-lethal heat stress in Arctic charr gill tissue (cGRASP32K). Validation analyses were based on robustness signatures CS0101i and CS0101u, and published signatures ES0013 (cGRASP44K), ES0012, ES0020, ES0021, ES0022 (GRASP16K), and ES0023 and ES0024 (cGRASP32K)

Signature	Data set	Species	Exposure duration	Tissue	Array platform	Signature
ES0012	Jeffries et al. [30], Chronic heat (survivors)	Sockeye salmon	Chronic	Gill	cGRASP16K	Publication Table S1A [30]
ES0013	Jeffries et al. [9], Chronic heat	Pink and Sockeye salmon	Chronic	Gill	cGRASP44K	Publication Table S4 [9]
ES0024	Quinn et al. [32], Chronic heat	Arctic charr	Chronic	Gill	cGRASP32K	Publication Table S1 [32]
ES0023	Quinn et al. [33], Heat tolerance	Arctic charr	Acute	Gill	cGRASP32K	Publication Table S2 [33] (union A-G; 444 features)
ES0020	Lewis et al. [34], 24 h post-treatment	Rainbow trout	Acute	Red blood cells	cGRASP16K	Publication Table 4 [34]
ES0021	Lewis et al. [34], 4 h post-treatment	Rainbow trout	Acute	Red blood cells	cGRASP16K	Publication Table 2 [34]
ES0022	Anttila et al. [12], Cardiac response to warming	Sockeye salmon	Chronic	Cardiac	cGRASP16K	Publication Table SI [12]
DS0101	2007 Robust limma signature (FDR < 0.01)	Sockeye salmon	Chronic	Gill	cGRASP44K	Derived in discovery analysis
DS0102	2008 Robust limma signature (FDR < 0.01)	Sockeye salmon	Chronic	Gill	cGRASP44K	Derived in discovery analysis
DS0103	2009 Robust limma signature (FDR < 0.01)	Pink salmon	Chronic	Gill	cGRASP44K	Derived in discovery analysis
CS0101i	Temperature 2007–2009 Discovery (FDR < 0.01; Intersection)	Pink and Sockeye salmon	Chronic	Gill	cGRASP44K	Derived in discovery analysis
CS0101u	Temperature 2007–2009 Discovery (FDR < 0.01; Union)	Pink and Sockeye salmon	Chronic	Gill	cGRASP44K	Derived in discovery analysis
EX0101a	2009 Temperature Gene Shaving Cluster 5 (“Shave5”)	Pink salmon	Chronic	Gill	cGRASP44K	Derived in discovery analysis
EX0102a	2007 Temperature Gene Shaving Cluster 4 (“Shave4”)	Sockeye salmon	Chronic	Gill	cGRASP16K	Derived in discovery analysis
EX0103a	2008 Temperature Combined sPCA2 + sPCA3	Sockeye salmon	Chronic	Gill	cGRASP44K	Derived in discovery analysis
DS0104	Robust limma signature (FDR < 0.01), Heat tolerance data	Arctic Charr	Acute	Gill	cGRASP32K	Derived in discovery analysis

Data from the Quinn et al. [33] study on heat tolerance in Arctic charr (cGRASP32K platform; GSE26306) was also used for discovery analysis, but samples were combined in a different way to determine a signature indicative of acute heat stress response rather than tolerance. Signature DS0104 was derived from robust limma analysis of this data set, contrasting 6 control samples at 6 °C with 12 samples held at 25 °C for 6–8 h (representing a mix of 6 heat-tolerant and 6 heat-intolerant fish). DS0104 was compared with discovered and published signatures, including signature ES0023, which represents the union of significant EST identifiers provided in Table S2 (A-G) in the Quinn et al. [33] publication.

### Literature mining

Broad scale manual text mining on thermal stress transcriptome published papers in fish was carried out to identify gene names regulated during thermal stress across all fish species, including salmonids. Moreover, the built-in text mining and knowledge extraction tool MedScan [90] was applied to mine abstracts and full papers for word associations with proteins. Using this approach, we could quickly identify biological relationships with key proteins (biomarkers) that the manuscript authors identified as most significantly associated with thermal stress, specifically focusing in on the fish literature, and could assess the breadth of thermal stress-based studies for which key biomarkers are shown to be activated. Unlike the microarray studies, these analyses were not restricted to specific tissues.

The terms e.g. ‘thermal stress’, ‘heat shock’, ‘cell stress’, ‘fish’, ‘gene expression’, ‘cold stress’, ‘heat shock protein’, ‘SERPINH1, ‘HSP90, ‘CIRBP, ‘FKBP10’, ‘PDIA4’, ‘PARK7’, ‘MAP3K14’, ‘COX1’, ‘MPDU1’, ‘EIF4A’, ‘EIF4E’, ‘NEK4’, ‘SEPW1’, ‘EEF2’, ‘SFRS2’, ‘UBE2Q2’, ‘SFRS9’, ‘KCT2’, ‘AP3S1’, ‘IDH3B’, TUBA1A ‘SCFD1’, ‘zgc:63572′ and ‘ZMYND11’ were included in literature mining. In total, 65 full papers concerning gene expression of thermal stress in fish and shellfish, including all seven studies in Table 6, were assessed. These papers studied gene expression in response to thermal stress in 30 fish species including 8 salmonids, two mussel species, one crab species and coral species.

### Selecting thermal stress biomarkers

Sequential criteria for gene selection were ranked as follows: (1) signatures on cGRASP44K that were found in the intersection of robust limma analysis based on high and low temperature samples in the 2007, 2008 and 2009 MGL Temperature data, and identified in Jeffries et al. 2014b and in at least two of 3 year-based unsupervised exploratory analyses of the MGL 2007–2009 Temperature data; (2) EST identifiers on GRASP16K from MGL and genes from GRASP16K/32 K published data that define candidate temperature biomarker signatures; (3) supported by comparison and robustness analyses across published studies; and (4) availability of the sequences for Pacific salmon in the internal MGL database and Genbank (National Center for Biotechnology Information; NCBI).

### Development of qRT-PCR assays for thermal stress across multiple salmon species

A relational database containing genome and microarray information of salmonids was produced for in-house large-scale qRT-PCR assay development. In this database, all microarray features and ESTs for GRASP, TRAITS, and SIQ microarray platforms were mapped to the Atlantic salmon Genome (assembly ICSASG_v2). This mapping was done using the STAR aligner [91], and the Atlantic Salmon Gene IDs associated with mapped locations were assigned using featureCounts from the Subread software package [92]. Unmapped sequences were annotated with Uniprot IDs from a merged version of the Swiss-Prot and TrEMBL databases using the blastx command in DIAMOND [93]. The database is organized at the level of Atlantic salmon Gene IDs and is available from the authors in addition to NCBI public repository chromosome numbers and accession numbers for protein and mRNA sequences.

In order to provide Pacific salmon sequence data for TaqMan assay development, transcriptomes from Chinook, coho, and sockeye salmon were enriched for microarray features using SureSelectXT (Agilent, Santa Clara, California, USA), and sequenced with IonTorrent (Thermo-Fisher Scientific). Briefly, six to eight individuals from each species were pooled to use as template, with a total of 750 ng total RNA per pool. The samples were fragmented, amplified, and purified using the Ion Total RNA-Seq kit V2 as per manufacturer’s instructions (Thermo-Fisher Scientific). 200 ng of the prepared library was carried into the SureSelect^XT^ workflow, using the SureSelect Target Enrichment System Kit for Sequencing on the Ion Proton (Agilent, Santa Clara, California, USA). The bait pool used consisted of all microarray features from the cGRASP 32 K and 44 K arrays. Sample libraries diluted to 100pM were prepared for sequencing on the Ion Torrent using the Ion PI HiQ Chef kit, and sequenced with the Ion PI Hi-Q Sequencing 200 Kit with an Ion PI Chip V3 as per manufacturer’s instructions (ThermoFisher Scientific). Following quality filtering, transcripts were assembled into contigs using the Trinity RNA-seq assembler for each species [94]. Contigs were then annotated and added to the database using the same mapping methods described above.

Gene IDs for selected candidate thermal stress biomarkers were retrieved from the database using the EST identifier from the microarray analyses. For studies using candidate genes, the Gene ID was retrieved using the accession number sequence and a nucleotide BLAST. For cases where an EST or sequence mapped to more than one gene ID, the gene ID that had more support from (1) NCBI repository salmonid sequence nucleotide BLAST alignments or (2) a larger number of Pacific salmon sequences mapped in the database, was initially selected. If there were limited Pacific salmon sequences in the database for a specific gene ID, sometimes additional sequences, in particular for Rainbow trout (*O. mykiss*), could be found using a nucleotide BLAST search of the nr/nt and EST databases.

Available sequences were aligned to the reference Atlantic salmon sequences of the specified Gene ID using Sequencher 5.4.5 (Gene Codes Corporation, Ann Arbor, MI) or MEGA 7.0.14 (http://www.megasoftware.net). Sequences from closely related gene transcripts (e.g. gene homologs or paralogs) were also included in the alignment. For qRT-PCR development, the focus was on sequence areas that were different among closely related gene transcripts yet similar across species. Candidate primers and TaqMan probes for the sequences were produced using Primer Express 3.0.1 (Thermo Fisher Scientific, Waltham, MA) with primer Tm between 58 and 60 °C, and probe Tm between 68 and 70 °C as default. Because we aimed to design assays that were gene specific, selected assays had probes that mismatched for one base pair in the last three bases at the 3′ end or for two or more base pairs. For some of the candidate biomarkers, two assays were designed for each gene ID for further efficiency testing and thermal stress biomarker validation.

### Quantitative PCR on the Fluidigm BioMark™ HD platform

To test the efficiency of the thermal stress TaqMan assays across species, cDNA from RNA extractions of pooled tissues from each of six salmonid species, including Chinook, pink, sockeye, coho, chum (*O. keta*) and Atlantic salmon (*Salmo salar*), were serially diluted from 1/5 to 1/625 in five dilutions. Specific Target Amplification (STA), required to attain high sensitivity with microfluidics quantitative PCR due to the 7 nl chamber size, was performed to enrich for targeted sequences within the pools, following the prescribed protocol. The 5 μl STA reaction contained 1.3 μl of cDNA/DNA, 1X TaqMan PreAmp master mix (Applied Biosystems, Foster City, CA, USA) and 0.2 μM of each of the primers (45 thermal host genes and 3 housekeeping genes). The 14-cycle STA program followed manufacturer’s instructions (Fluidigm Corporation, South San Francisco, CA, USA). Upon completion of the STA, excess primers were removed by treating with Exo-SAP-IT™ (Affymetrix, Santa Clara, CA) according to manufacturer’s instructions and then diluted 1/5 in DNA re-suspension buffer (Teknova, Hollister, CA).

For efficiency testing, a 48.48 gene expression Dynamic Array, which using microfluidics allows 48 samples to be simultaneously assessed across 48 TaqMan assays, was applied, systematically combined into 2304 individual reactions. Amplification plots were scored using the Real-Time PCR Analysis Software (Fluidigm) and data from multiple dynamic arrays were combined using GenEx software (http://www.multid.se/). PCR efficiencies for each assay were calculated using the eq. E% = (10 ^1/slope^ - 1) × 100, where the slope was estimated plotting the Ct over the serial dilutions of cDNA. The assays that worked across all salmon species and showed a good efficiency (ideally between 0.9–1.1) were selected for thermal stress biomarker validation.

For validation of selected thermal stress biomarkers, we applied the BioMark system analysis to cDNA from adult sockeye salmon temperature challenge studies conducted in 2007/2008 [9, 30], and juvenile Chinook salmon challenged in 2017/18. Sockeye salmon studies were carried out on return-migrating adult sockeye salmon from different populations within the Fraser River (Lower Adams and Chilko River populations in 2007, and lower Harrison in 2008), British Columbia, Canada. In each year, fish were collected by beach seine from the main stem of the Fraser River, and transported to the Fisheries and Oceans Canada Cultus Lake Salmon Research Laboratory, near Chilliwack, British Columbia, Canada, where they were randomly distributed among 8000-L aerated tanks at 10–12 °C. The tank water temperatures were subsequently raised at a rate of 2–2.5 °C day− 1 until the test temperatures of 13 or 14 °C (cool treatment), 16 °C (moderate thermal stress, only in 2008) and 19 °C (thermal stress treatment) were reached. After 7 days in 2007 and 5 days in 2008, small pieces of gill tissue were non-lethally sampled from survivor fish in both temperature groups in 2007 and three temperature groups in 2008 to determine the effect of water temperature on gene expression [9, 30]. Moreover, moribund fish kept in both temperatures in 2007 were sampled for gill tissue after 7 days and then euthanized in an overdose of buffered TMS. Chinook salmon samples were part of a multi-stressor challenge study conducted in 2017/2018; here, we restrict our presentation to the thermal challenge conducted in freshwater. Briefly, sub-yearling ocean-type Chinook salmon juveniles were acquired from Big Qualicum River Hatchery, Qualicum Beach, British Columbia, Canada and transported to the Pacific Biological Station, and were reared in communal circular tanks supplied with freshwater (14 °C), until used in the experimental trials. Four trials were conducted, including pre-smolt, smolt, and de-smolt stages of development. Juveniles were moved to the 30 L experimental tanks for acclimation and temperature was raised at a rate of 2 °C day− 1 until the test temperature of 18 °C (thermal stress treatment) was reached. After 6 days of exposure to 14 and 18 °C, juveniles were euthanized in an overdose of buffered TMS (250 mg L^− 1^) using water of the same temperature, and gill tissue was sampled from survivor and moribund fish kept at both temperatures. Gill samples were immediately flash frozen in liquid nitrogen and stored at − 80 °C until analysis. Specific details of the fish populations studied and the temperature treatment experiments for each year are provided in Table 7.

**Table 7:** Details of experimental design for the sockeye and Chinook salmon held at a warm or cool temperature in three different temperature-holding experiments conducted in 2007–2008 and 2017–2018

Year	Survival	Treatment duration (days)	Temperature (°C)	N discovery	N validation
Sockeye salmon					
2007	Survivor	7	14	8	6
		7	19	13	11
	Moribund	7	14	–	10
		7	19	–	13
2008	Survivor	5	13	14	14
		5	16	–	13
		5	19	19	13
Chinook salmon					
2017–2018	Survivor	6	14	–	20
		6	18	–	16
	Moribund	6	14	–	6
		6	18	–	5
Total				54	127

While a portion of the samples used for biomarker validation were also part of our discovery analysis (i.e., with associated microarray data), additional samples that were not part of the microarray studies were also included. All 40 gill samples of 2007 sockeye salmon used for biomarker validation were part of the Jeffries et al. [9] study. The subset of 17 sockeye salmon survivors from the 2007 temperature challenge were used for both discovery and validation analyses. The remaining 23 gill samples from 2007 moribund sockeye salmon kept at 13 °C and 19 °C were not part of the discovery analysis, but used for the first time in validation analysis. Moreover, 25 gill samples from 2008 sockeye salmon kept in 13 °C and 19 °C were also used for both discovery and validation analyses. Thirteen gill samples from 2008 sockeye salmon kept at 16 °C were used for the first time in validation analysis to observe the effect of chronic moderate thermal stress in salmonids (Table 7). None of the 47 Chinook salmon samples used in validation analysis were part of the discovery analysis.

RNA was quantitated and normalized to 62.5 ng/μl with a Biomek NXP (Beckman-Coulter, Mississauga, ON, Canada) automated liquid-handling instrument. RNA (1 μg) was reverse transcribed into cDNA using the superscript VILO master mix kit (Invitrogen, Carlsbad, CA). The cDNA was then used as template for STA as described above. The 96.96 gene expression dynamic array (Fluidigm Corporation, CA, USA) was applied and generally followed Miller et al. (2016). QRT-PCR data were analysed with Real-Time PCR Analysis 3 Software (Fluidigm Corporation, CA, USA).

Prior to statistical analysis, amplification efficiency (E) was determined for each target gene as described above. The expression of target genes relative to the reference genes was calculated by the 2^–ΔΔC^_T_ method as convenient way to analyze the relative changes in gene expression [95]. All qRT-PCR data were log2-transformed and the homogeneity of variances and normality were assessed by Bartlett’s and Kolmogorov–Smirnov tests, respectively. Differences in gene expression data between different thermal treatments were analyzed by a one-way analysis of variance (ANOVA), followed by a Tukey’s HSD post hoc analysis for multiple comparisons. Statistical analyses were conducted with SigmaPlot (version 11), and the graphs were constructed in R 3.1.2 (https://cran.r-project.org/).

## Additional files

Additional file 1: **Table S1.** A 3-set comparison of 69 consistently higher expressed EST identifiers and 70 consistently lower expressed identifiers in the warmer temperatures in robust limma analyses (FDR < 0.01) for the 2007 sockeye, 2008 sockeye and 2009 pink salmon data sets. (DOC 204 kb)

Additional file 2: **Figure S1.** Shown are a heatmap (left) and PCA plot (right) of 98 samples for 9 features returned by Gene Shaving applied to the union of robust limma (FDR < 0.01) signatures (5254 features) for separate discovery analysis of the 2007 sockeye, 2008 sockeye and 2009 pink salmon data sets. (JPG 2421 kb)

Additional file 3: **Figure S2.** Shown is a heatmap (left) and PCA plot (right) of 44 pink samples for 21 features returned by Gene Shaving applied to the filtered pink salmon 29,615-feature data set. (JPG 2432 kb)

Additional file 4: **Figure S3.** Shown is a heatmap (left) and PCA plot (right) of 21 sockeye salmon samples (2007) for 98 features returned by Gene Shaving applied to the filtered 2007 sockeye salmon 30,072-feature data set. (JPG 2470 kb)

Additional file 5: **Figure S4.** Shown are sPCA scatter plots (left) and heatmaps for sPCA component 2 (top right) and component 3 (bottom right) for 33 sockeye salmon samples (2008). 100 features were returned for each of the sPCA components when sPCA was applied to the filtered 2008 sockeye salmon 29,657-feature data set. (JPG 3231 kb)

Additional file 6: **Table S2.** Gene summary table for 153 EST identifiers on cGRASP44K that define candidate temperature biomarker signature CTS001. The signature is mainly composed of 139 identifiers that were found in the intersection of robust limma analysis based on high and low temperature samples in the 2007, 2008 and 2009 MGL Temperature data set (CS0101i). Four additional features were found in Jeffries et al. 2014b published 49-identifier list and an additional ten EST identifiers were found in at least two of 3 year-based unsupervised exploratory analyses of the MGL 2007–2009 Temperature data. (DOC 183 kb)

## Abbreviations

AP3S1: AP-3 complex subunit sigma-1
CIRBP: Cold-inducible RNA-binding protein B
COX6B1: Cytochrome C oxidase
EEF2: Eukaryotic translation elongation factor 2
EIF4A2: Eukaryotic translation initiation factor 4A, isoform 2
EIF4ENIF1: Eukaryotic translation initiation factor 4E nuclear import factor 1
FKBP10: FK506-binding protein 10 precursor
GEO: Gene Expression Omnibus
HSC71: Heat shock cognate 70 kDa protein
HSP70: Heat shock 70 kDa protein
HSP90AA1: Heat shock protein HSP 90-alpha
HSP90AB1: Heat shock protein 90 kDa alpha (cytosolic) class B member 1
IDH3B: Isocitrate dehydrogenase 3 (NAD+) beta
KCT2: Keratinocytes-associated transmembrane protein 2 precursor
MAP3K14: Mitogen-activated protein kinase kinase kinase 14
MGL: Molecular Genetics Laboratory
MPDU1: Mannose-P-dolichol utilization defect 1 protein
NEK4: Serine/threonine-protein kinase Nek4
PARK7: Parkinson disease (autosomal recessive, early onset) 7 (Protein DJ-1)
PCA: Principal Components Analysis
PDIA4: Protein disulfide-isomerase A4 precursor
qRT-PCR: Quantitative Real-Time Polymerase Chain Reaction
SCFD1: Sec1 family domain-containing protein 1
SEPW1: Selenoprotein W
SERPINH1: Serpin H1 precursor
SFRS2: Splicing factor, arginine/serine-rich 2
SFRS9: Splicing factor, arginine/serine-rich 9
TUBA1A: Tubulin alpha-1A chain
UBE2Q2: Ubiquitin-conjugating enzyme E2 Q2
zgc:63572: Transmembrane protein 185
ZMYND11: Zinc finger MYND domain-containing protein 11
