# Supplementary material for: Developing specific molecular biomarkers for thermal stress in salmonids
Source: BMC Genomics. 2018 Oct 16;19:749. doi: 10.1186/s12864-018-5108-9 (PMC6192343; doi:10.1186/s12864-018-5108-9)
Supplement: Supplementary file 1 — Table S1. A 3-set comparison of 69 consistently higher expressed EST identifiers and 70 consistently lower expressed identifiers in the warmer temperatures in robust limma analyses (FDR < 0.01) for the 2007 sockeye, 2008 sockeye and 2009 pink salmon data sets. (DOC 204 kb) [file 12864_2018_5108_MOESM1_ESM.doc]

Table S1. A 3-set comparison of 69 consistently higher expressed EST identifiers and 70 consistently lower expressed identifiers in the warmer temperatures in robust limma analyses (FDR<0.01) for the 2007 sockeye, 2008 sockeye and 2009 pink salmon data sets.

|  | **EST identifiers** | **Gene Name** | **2007 Sockeye 14 vs. 19 ºC** | | **2008 Sockeye 13 vs. 19 ºC** | | **2009 Pink 13 vs. 19 ºC** | |
| --- | --- | --- | --- | --- | --- | --- | --- | --- |
|  |  |  | **Mean 14 ºC** | **Mean 19 ºC** | **Mean 13 ºC** | **Mean 19 ºC** | **Mean 13 ºC** | **Mean 19 ºC** |
| 1 | C044R036 | Serpin H1 precursor | -1.53 | 1.67 | -2.59 | 0.73 | -1.91 | 1.05 |
| 2 | C205R085 | Serpin H1 precursor | -1.57 | 1.95 | -2.91 | 0.62 | -2.32 | 0.60 |
| 3 | C236R132 | Serpin H1 precursor | -1.19 | 1.80 | -2.37 | 0.92 | -2.12 | 0.67 |
| 4 | C009R155 | Serpin H1 precursor | -1.54 | 1.55 | -2.61 | 0.77 | -1.94 | 0.81 |
| 5 | C265R019 | Serpin H1 precursor | -1.30 | 1.62 | -2.26 | 0.71 | -2.00 | 0.71 |
| 6 | C020R155 | Heat shock protein HSP 90-beta | -2.68 | -0.31 | -2.08 | -0.75 | -0.80 | 1.68 |
| 7 | C015R121 | Heat shock protein HSP 90-alpha | -1.49 | 1.13 | -1.11 | 0.14 | -1.35 | 1.10 |
| 8 | C198R091 | Heat shock protein HSP 90-alpha 1 | -0.96 | 1.13 | -1.11 | 0.44 | -1.04 | 0.96 |
| 9 | C026R101 | DnaJ homolog subfamily A member 4 | -0.35 | 1.03 | -0.71 | 0.13 | -0.94 | 0.75 |
| 10 | C169R048 | Heat shock cognate 71 kDa protein | -1.18 | 0.24 | -1.89 | -0.86 | -0.61 | 0.87 |
| 11 | C113R139 | Guanidinoacetate N-methyltransferase | -1.88 | 0.10 | -1.14 | 0.29 | -1.03 | 0.40 |
| 12 | C182R027 | Heat shock protein HSP 90-alpha | -0.54 | 0.88 | -0.37 | 0.46 | -0.62 | 0.74 |
| 13 | C204R073 | Zinc finger and BTB domain-containing protein 46 | -0.04 | 0.85 | -0.02 | 0.95 | -0.68 | 0.59 |
| 14 | C075R011 | Tubulin alpha-1A chain | 0.34 | 1.52 | -0.49 | 1.04 | -0.79 | 0.48 |
| 15 | C134R037 | Heat shock 70 kDa protein 4L | -0.53 | 0.55 | -1.37 | -0.65 | -0.30 | 0.93 |
| 16 | C014R149 | Mitogen-activated protein kinase kinase kinase 14 | -0.69 | 0.32 | -0.75 | 0.12 | -0.25 | 0.97 |
| 17 | C060R057 | Mitogen-activated protein kinase kinase kinase 14 | -0.86 | 0.09 | -1.00 | -0.16 | -0.31 | 0.89 |
| 18 | C051R015 | Granzyme-like protein 1 precursor | -1.80 | -0.72 | -2.45 | -1.54 | -0.06 | 1.07 |
| 19 | C240R154 | Eukaryotic translation initiation factor 4E transporter | 0.34 | 1.39 | 0.32 | 1.26 | -1.46 | -0.35 |
| 20 | C062R114 | Tubulin alpha-1A chain | 0.17 | 1.26 | -0.41 | 1.14 | -0.94 | 0.15 |
| 21 | C036R054 | Tubulin alpha-1A chain | 0.06 | 1.27 | -0.32 | 1.23 | -0.84 | 0.18 |
| 22 | C131R020 | 60S ribosomal protein L32 | -0.10 | 1.16 | 0.01 | 1.08 | -1.32 | -0.32 |
| 23 | C114R035 | "Splicing factor, arginine/serine-rich 6" | -0.04 | 0.67 | -0.42 | 0.44 | -0.47 | 0.52 |
| 24 | C158R169 | 60S ribosomal protein L36a | 0.53 | 1.60 | 0.56 | 1.63 | -2.47 | -1.57 |
| 25 | C246R153 | Protein DJ-1 | 0.48 | 1.53 | 0.17 | 1.16 | -0.90 | -0.01 |
| 26 | C096R117 | Eukaryotic initiation factor 4A-II | 0.21 | 0.98 | 0.22 | 0.91 | -0.84 | 0.03 |
| 27 | C213R047 | Glycine N-acyltransferase | -0.55 | 0.58 | 0.01 | 0.67 | -0.54 | 0.31 |
| 28 | C035R144 | Selenoprotein W | -0.18 | 0.80 | -0.11 | 0.52 | -0.11 | 0.71 |
| 29 | C046R166 | Heterogeneous nuclear ribonucleoprotein G | 0.14 | 0.78 | -0.13 | 0.59 | -0.41 | 0.40 |
| 30 | C052R159 | Selenoprotein W | -0.36 | 0.56 | -0.44 | 0.55 | -0.14 | 0.65 |
| 31 | C020R006 | Lysozyme g | 0.05 | 0.87 | 0.33 | 1.10 | -1.31 | -0.52 |
| 32 | C071R163 | "Splicing factor, arginine/serine-rich 7" | 0.19 | 0.96 | -0.32 | 0.38 | -0.32 | 0.45 |
| 33 | C193R155 | Cytochrome c oxidase subunit VIb isoform 1 | -1.49 | -0.19 | -0.62 | 0.46 | -0.16 | 0.60 |
| 34 | C051R114 | UNKNOWN | -0.30 | 0.32 | -0.22 | 0.44 | -0.48 | 0.28 |
| 35 | C194R063 | Cysteine-rich with EGF-like domain protein 1 precursor | -0.18 | 0.62 | -0.09 | 0.48 | -0.14 | 0.61 |
| 36 | C263R094 | Protein disulfide-isomerase A4 precursor | -0.04 | 0.64 | 0.03 | 0.44 | -0.58 | 0.14 |
| 37 | C142R109 | Selenoprotein W | -0.28 | 0.62 | -0.33 | 0.60 | -0.15 | 0.57 |
| 38 | C119R133 | Phosphatidylethanolamine-binding protein 1 | 0.30 | 0.77 | 0.07 | 0.39 | -0.73 | -0.02 |
| 39 | C258R108 | Selenoprotein W | -0.28 | 0.60 | -0.43 | 0.46 | -0.09 | 0.59 |
| 40 | C092R059 | Eukaryotic initiation factor 4A-II | 0.10 | 0.79 | 0.10 | 0.71 | -0.53 | 0.14 |
| 41 | C084R135 | "Splicing factor, arginine/serine-rich 7" | 0.14 | 0.59 | -0.14 | 0.38 | -0.16 | 0.51 |
| 42 | C071R082 | CD226 antigen precursor | -0.66 | -0.08 | -0.50 | 0.02 | -0.23 | 0.43 |
| 43 | C037R148 | UNKNOWN | 0.41 | 1.12 | 0.63 | 1.20 | -1.47 | -0.81 |
| 44 | C031R011 | "Splicing factor, arginine/serine-rich 7" | 0.08 | 0.65 | -0.19 | 0.48 | -0.21 | 0.43 |
| 45 | C101R077 | Serpin H1 precursor | -0.11 | 0.53 | -0.18 | 0.22 | -0.13 | 0.49 |
| 46 | C065R036 | "Acyl-CoA synthetase family member 2, mitochondrial precursor" | 0.18 | 0.56 | -0.03 | 0.44 | -0.16 | 0.46 |
| 47 | C057R105 | UNKNOWN | -0.30 | 0.29 | -0.23 | 0.34 | 0.16 | 0.77 |
| 48 | C107R106 | "Splicing factor, arginine/serine-rich 1" | 0.26 | 0.71 | -0.15 | 0.32 | -0.21 | 0.39 |
| 49 | C260R050 | sialic acid acetylesterase | -0.15 | 0.26 | -0.17 | 0.26 | -0.26 | 0.30 |
| 50 | C135R033 | Host cell factor 2 | 0.68 | 1.07 | 0.55 | 0.95 | -1.14 | -0.59 |
| 51 | C097R152 | G protein pathway suppressor 2 | -0.08 | 0.49 | -0.09 | 0.41 | -0.32 | 0.21 |
| 52 | C155R164 | Selenoprotein W | -0.44 | 0.46 | -0.56 | 0.47 | 0.06 | 0.58 |
| 53 | C163R137 | Serine/threonine-protein kinase Nek4 | 0.61 | 1.26 | 0.24 | 1.07 | -1.14 | -0.66 |
| 54 | C257R028 | Stathmin | 0.73 | 1.26 | 0.04 | 0.59 | -0.81 | -0.33 |
| 55 | C088R019 | Histone H2A | -0.07 | 0.63 | 0.23 | 0.63 | -0.48 | -0.01 |
| 56 | C101R112 | Heat shock protein HSP 90-beta | -0.70 | 0.02 | -0.59 | -0.23 | -0.77 | -0.30 |
| 57 | C117R126 | UNKNOWN | -0.04 | 0.46 | 0.24 | 0.72 | -0.53 | -0.13 |
| 58 | C199R092 | UNKNOWN | -0.32 | 0.23 | -0.30 | 0.18 | -0.15 | 0.23 |
| 59 | C011R144 | Thymosin beta-a | -1.97 | -0.68 | -1.22 | -0.36 | 0.32 | 0.70 |
| 60 | C078R023 | Protein DEK | 0.14 | 0.69 | 0.36 | 1.03 | -0.59 | -0.23 |
| 61 | C050R032 | "Splicing factor, arginine/serine-rich 7" | -0.21 | 0.30 | -0.24 | 0.17 | -0.03 | 0.29 |
| 62 | C225R163 | Carbonic anhydrase 6 precursor | -0.71 | -0.04 | -0.03 | 0.53 | -0.03 | 0.29 |
| 63 | C159R065 | "NADH dehydrogenase [ubiquinone] iron-sulfur protein 7, mitochondrial precursor" | 0.66 | 0.99 | 0.39 | 1.13 | -0.90 | -0.59 |
| 64 | C227R013 | DPH3 homolog | -0.01 | 0.49 | 0.34 | 0.70 | -0.19 | 0.10 |
| 65 | C262R084 | Proteasome activator complex subunit 3 | 0.44 | 1.13 | 0.15 | 0.73 | -0.99 | -0.71 |
| 66 | C018R129 | Delta-aminolevulinic acid dehydratase | -0.85 | -0.37 | -0.84 | -0.48 | 0.26 | 0.53 |
| 67 | C242R131 | Chromobox protein homolog 1 | -0.32 | 0.25 | -0.25 | 0.08 | 0.03 | 0.30 |
| 68 | C241R021 | "Alpha-N-acetylgalactosaminide alpha-2,6-sialyltransferase 6" | -0.76 | -0.07 | -0.54 | 0.05 | 0.15 | 0.40 |
| 69 | C187R026 | "Splicing factor, arginine/serine-rich 1" | 0.12 | 0.92 | 0.14 | 0.71 | -0.02 | 0.22 |
| 70 | C101R159 | Transmembrane emp24 domain-containing protein 2 precursor | -0.33 | -0.77 | -0.43 | -1.01 | 0.29 | 0.02 |
| 71 | C185R026 | RNA 3'-terminal phosphate cyclase | -0.27 | -0.62 | -0.18 | -0.52 | 0.05 | -0.25 |
| 72 | C058R073 | BTB/POZ domain-containing protein KCTD6 | -0.06 | -0.35 | 0.16 | -0.21 | 0.00 | -0.34 |
| 73 | C142R150 | Cleft lip and palate transmembrane protein 1 homolog | 0.55 | 0.01 | 0.40 | -0.02 | -0.09 | -0.45 |
| 74 | C230R135 | Vacuolar ATP synthase 16 kDa proteolipid subunit | 0.28 | -0.02 | 0.46 | 0.16 | -0.30 | -0.66 |
| 75 | C115R159 | Oxysterol-binding protein-related protein 11 | 0.94 | 0.51 | 0.91 | 0.39 | -0.23 | -0.61 |
| 76 | C244R146 | Protein NDRG2 | -0.17 | -1.01 | -0.76 | -1.35 | 0.28 | -0.14 |
| 77 | C025R140 | DCN1-like protein 1 | -0.79 | -1.33 | -0.72 | -1.07 | 0.51 | 0.08 |
| 78 | C182R024 | UNKNOWN | 0.11 | -0.46 | 0.19 | -0.30 | -0.14 | -0.58 |
| 79 | C102R083 | Elongation factor 2 | 0.11 | -1.49 | 0.50 | -0.86 | -0.31 | -0.80 |
| 80 | C174R067 | Annexin A2 | 0.01 | -0.93 | -0.52 | -1.28 | 0.36 | -0.12 |
| 81 | C239R009 | Sialin | 0.01 | -0.65 | 0.34 | -0.14 | 0.13 | -0.38 |
| 82 | C119R035 | Transcription factor 12 | 0.09 | -0.57 | 0.35 | -0.11 | -0.12 | -0.65 |
| 83 | C007R028 | Mannose-P-dolichol utilization defect 1 protein | -0.81 | -1.69 | -0.95 | -1.53 | 0.31 | -0.24 |
| 84 | C135R059 | Wiskott-Aldrich syndrome protein family member 2 | 0.41 | -0.49 | 0.13 | -0.46 | -0.02 | -0.57 |
| 85 | C102R089 | Keratinocytes-associated transmembrane protein 2 precursor | 0.96 | 0.31 | 1.07 | 0.51 | -0.70 | -1.28 |
| 86 | C066R115 | "NADPH:adrenodoxin oxidoreductase, mitochondrial precursor" | 0.30 | -0.30 | 0.21 | -0.35 | 0.11 | -0.47 |
| 87 | C088R032 | Protein CutA homolog precursor | 0.21 | -0.53 | -0.12 | -0.72 | 0.10 | -0.49 |
| 88 | C202R096 | Brain protein 44-like protein | 0.77 | -0.13 | 0.49 | -0.25 | -0.06 | -0.66 |
| 89 | C072R039 | UNKNOWN | -0.79 | -1.56 | -1.10 | -1.67 | 0.71 | 0.11 |
| 90 | C120R146 | Hypoxia-inducible factor 1 alpha | -0.85 | -1.33 | -0.75 | -1.20 | 0.43 | -0.18 |
| 91 | C128R089 | Acid trehalase-like protein 1 | -0.24 | -1.07 | -0.39 | -1.09 | 0.58 | -0.05 |
| 92 | C221R165 | Major facilitator superfamily domain-containing protein 7 | 0.23 | -0.26 | 0.34 | -0.12 | 0.49 | -0.17 |
| 93 | C015R067 | Transmembrane protein 16A | -0.06 | -0.80 | -0.36 | -0.94 | 0.35 | -0.32 |
| 94 | C070R119 | FK506-binding protein 10 precursor | 0.00 | -0.70 | -0.25 | -0.87 | -0.05 | -0.73 |
| 95 | C018R140 | Probable peptidyl-tRNA hydrolase 2 | -0.46 | -1.04 | -0.25 | -0.92 | 0.51 | -0.17 |
| 96 | C164R154 | Exocyst complex component 3-like protein 2 | -0.63 | -1.41 | -0.96 | -1.71 | 0.78 | 0.10 |
| 97 | C192R015 | UNKNOWN | -0.54 | -1.32 | -0.61 | -1.02 | -0.17 | -0.86 |
| 98 | C014R004 | FK506-binding protein 10 precursor | 0.38 | -0.22 | 0.00 | -0.65 | 0.06 | -0.64 |
| 99 | C131R114 | "Glycogen phosphorylase, muscle form" | 0.10 | -0.60 | -0.53 | -1.04 | 0.32 | -0.38 |
| 100 | C036R039 | EH domain-containing protein 1 | 0.00 | -1.02 | -0.12 | -0.99 | 0.46 | -0.27 |
| 101 | C045R100 | Sodium/potassium-transporting ATPase subunit alpha-1 precursor | -0.01 | -0.77 | 0.07 | -0.71 | 0.02 | -0.74 |
| 102 | C061R023 | AP-3 complex subunit sigma-1 | 0.23 | -0.50 | 0.33 | -0.35 | -0.06 | -0.84 |
| 103 | C217R107 | UNKNOWN | 0.31 | -0.45 | 0.40 | -0.19 | 0.08 | -0.71 |
| 104 | C194R143 | UNKNOWN | 0.64 | -0.17 | 0.87 | 0.10 | -0.59 | -1.46 |
| 105 | C097R045 | Histone-arginine methyltransferase CARM1 | 0.03 | -1.00 | -0.05 | -1.03 | -0.20 | -1.07 |
| 106 | C125R081 | "Isocitrate dehydrogenase [NAD] subunit beta, mitochondrial precursor" | -0.84 | -1.69 | -0.99 | -1.78 | 0.38 | -0.49 |
| 4107 | C089R055 | Ubiquitin-conjugating enzyme E2 Q2 | 0.74 | -0.04 | 0.83 | 0.03 | -0.23 | -1.11 |
| 108 | C160R143 | Transmembrane protein 185-like | -0.35 | -1.38 | -0.28 | -1.23 | 0.34 | -0.56 |
| 109 | C254R016 | "Splicing factor, arginine/serine-rich 2" | 0.16 | -0.67 | 0.39 | -0.36 | -0.07 | -0.97 |
| 110 | C140R056 | Sodium/potassium-transporting ATPase subunit alpha-1 precursor | -0.19 | -0.93 | -0.13 | -0.85 | -0.02 | -0.93 |
| 111 | C222R071 | Membrane-associated transporter protein | -0.21 | -1.27 | -0.02 | -1.27 | 0.28 | -0.65 |
| 112 | C174R079 | "Splicing factor, arginine/serine-rich 2" | 0.24 | -0.79 | 0.23 | -0.61 | -0.07 | -1.02 |
| 113 | C084R083 | "Splicing factor, arginine/serine-rich 2" | -0.01 | -1.04 | 0.00 | -0.78 | 0.11 | -0.86 |
| 114 | C154R080 | FK506-binding protein 10 precursor | -0.04 | -1.05 | -0.32 | -1.21 | -0.18 | -1.17 |
| 115 | C145R015 | "Tubulin alpha chain, testis-specific" | -0.15 | -1.07 | -0.02 | -0.76 | -0.23 | -1.22 |
| 116 | C078R152 | "Splicing factor, arginine/serine-rich 2" | -0.22 | -1.30 | 0.10 | -0.84 | 0.01 | -1.00 |
| 117 | C057R065 | Uncharacterized protein C18orf8 | -0.76 | -1.64 | -0.56 | -1.56 | 0.39 | -0.63 |
| 118 | C251R142 | Cold-inducible RNA-binding protein | 0.50 | -0.75 | 0.43 | -0.38 | -0.25 | -1.28 |
| 119 | C036R063 | "Splicing factor, arginine/serine-rich 2" | 0.65 | -0.41 | 0.73 | -0.04 | -0.20 | -1.24 |
| 120 | C040R109 | UNKNOWN | -0.45 | -1.33 | -0.57 | -1.25 | 0.73 | -0.33 |
| 121 | C090R103 | Membrane-associated transporter protein | -0.26 | -1.61 | 0.00 | -1.79 | 0.25 | -0.81 |
| 122 | C204R003 | Class B basic helix-loop-helix protein 5 | -0.53 | -1.41 | -0.89 | -1.65 | 0.71 | -0.38 |
| 123 | C187R068 | "Splicing factor, arginine/serine-rich 2" | -0.61 | -1.60 | -0.41 | -1.22 | -0.09 | -1.19 |
| 124 | C240R152 | UNKNOWN | 1.48 | -0.01 | 1.13 | -0.02 | -0.55 | -1.67 |
| 125 | C165R115 | FK506-binding protein 10 precursor | -0.20 | -1.31 | -0.58 | -1.31 | -0.12 | -1.28 |
| 126 | C026R122 | Cold-inducible RNA-binding protein | -1.28 | -1.65 | -1.18 | -1.55 | 0.19 | -0.98 |
| 127 | C205R010 | Sec1 family domain-containing protein 1 | -0.33 | -1.13 | -0.13 | -0.91 | 0.25 | -0.92 |
| 128 | C067R011 | FK506-binding protein 10 precursor | -0.16 | -1.29 | -0.52 | -1.40 | -0.09 | -1.31 |
| 129 | C171R003 | FK506-binding protein 10 precursor | -0.21 | -1.28 | -0.50 | -1.34 | -0.04 | -1.30 |
| 130 | C054R085 | UNKNOWN | -0.05 | -1.36 | -0.35 | -1.05 | 0.57 | -0.71 |
| 131 | C107R130 | Cold-inducible RNA-binding protein | -0.18 | -1.83 | -0.11 | -1.74 | -0.20 | -1.52 |
| 132 | C124R099 | FK506-binding protein 10 precursor | -0.06 | -1.11 | -0.15 | -1.13 | 0.13 | -1.27 |
| 133 | C002R034 | "Troponin C, slow skeletal and cardiac muscles" | 1.11 | 0.03 | 0.80 | -0.51 | 0.02 | -1.44 |
| 134 | C262R107 | Elongation factor 2 | -0.06 | -1.00 | 0.63 | -0.87 | 0.25 | -1.22 |
| 135 | C074R053 | "Troponin C, slow skeletal and cardiac muscles" | 1.82 | 0.45 | 1.40 | 0.13 | -0.90 | -2.48 |
| 136 | C142R097 | "Troponin C, slow skeletal and cardiac muscles" | 1.53 | -0.06 | 0.84 | -0.42 | 0.10 | -1.58 |
| 137 | C074R026 | "Splicing factor, arginine/serine-rich 9" | -0.50 | -1.97 | -0.06 | -1.60 | 0.25 | -1.44 |
| 138 | C025R120 | "Splicing factor, arginine/serine-rich 9" | -0.22 | -1.85 | 0.27 | -1.30 | 0.32 | -1.38 |
| 139 | C190R138 | Zinc finger MYND domain-containing protein 11 | -0.75 | -1.62 | -0.42 | -1.38 | 0.26 | -1.44 |
