# Supplementary figures and images for: Developing specific molecular biomarkers for thermal stress in salmonids
Source: BMC Genomics. 2018 Oct 16;19:749. doi: 10.1186/s12864-018-5108-9 (PMC6192343; doi:10.1186/s12864-018-5108-9)

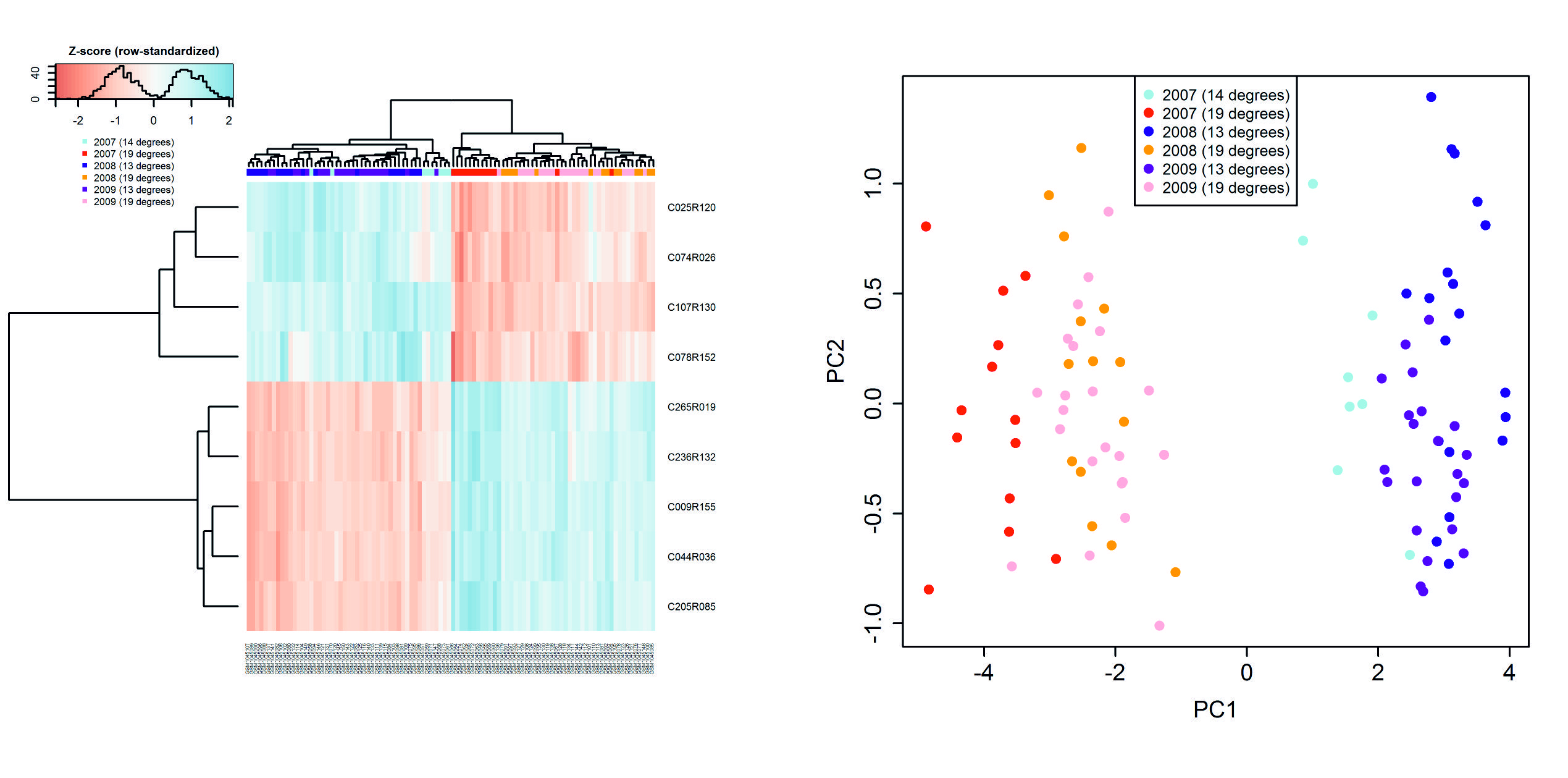

Supplement: Supplementary file 2 — Figure S1. Shown are a heatmap (left) and PCA plot (right) of 98 samples for 9 features returned by Gene Shaving applied to the union of robust limma (FDR < 0.01) signatures (5254 features) for separate discovery analysis of the 2007 sockeye, 2008 sockeye and 2009 pink salmon data sets. (JPG 2421 kb) [file 12864_2018_5108_MOESM2_ESM.jpg]

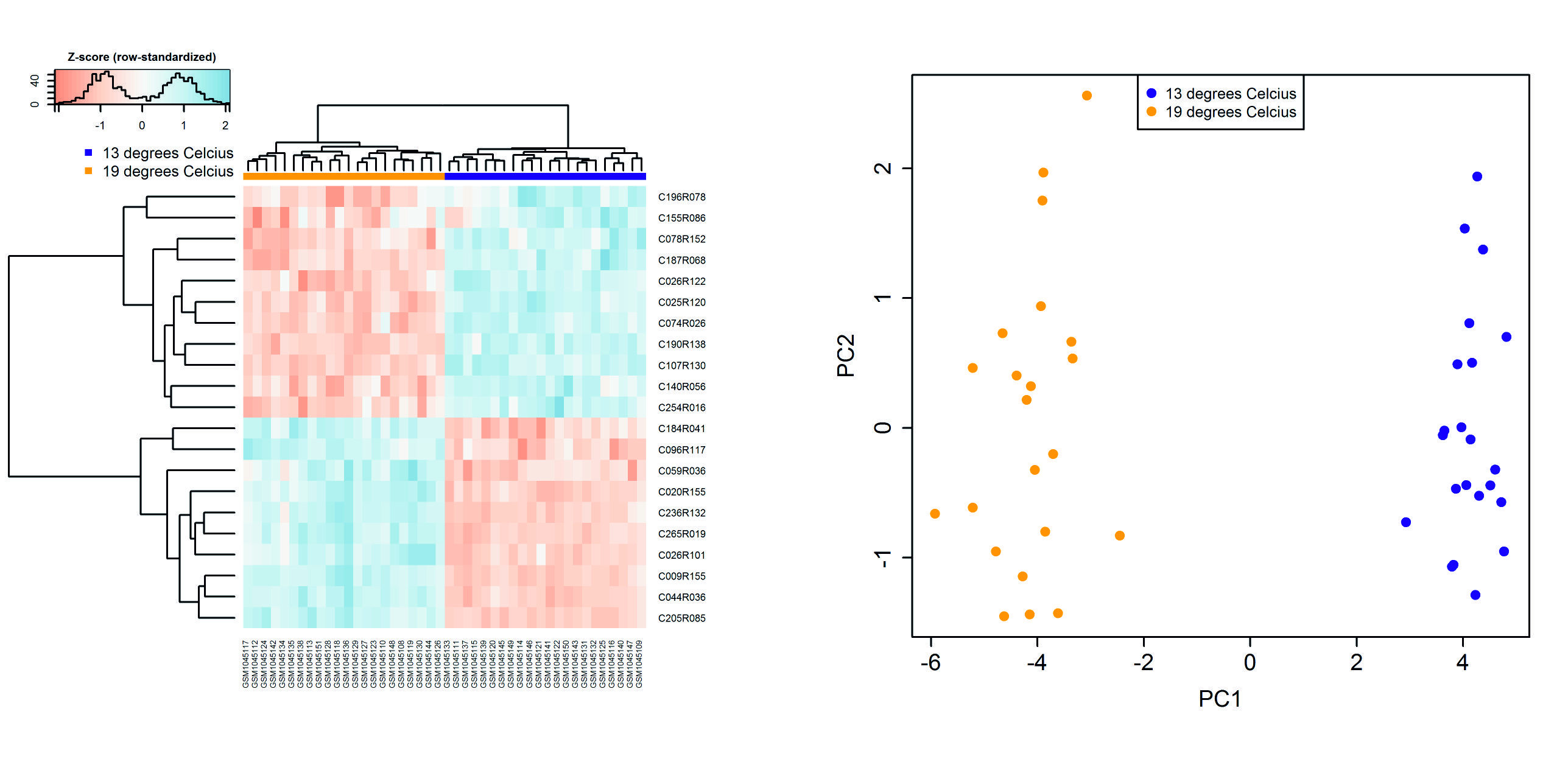

Supplement: Supplementary file 3 — Figure S2. Shown is a heatmap (left) and PCA plot (right) of 44 pink samples for 21 features returned by Gene Shaving applied to the filtered pink salmon 29,615-feature data set. (JPG 2432 kb) [file 12864_2018_5108_MOESM3_ESM.jpg]

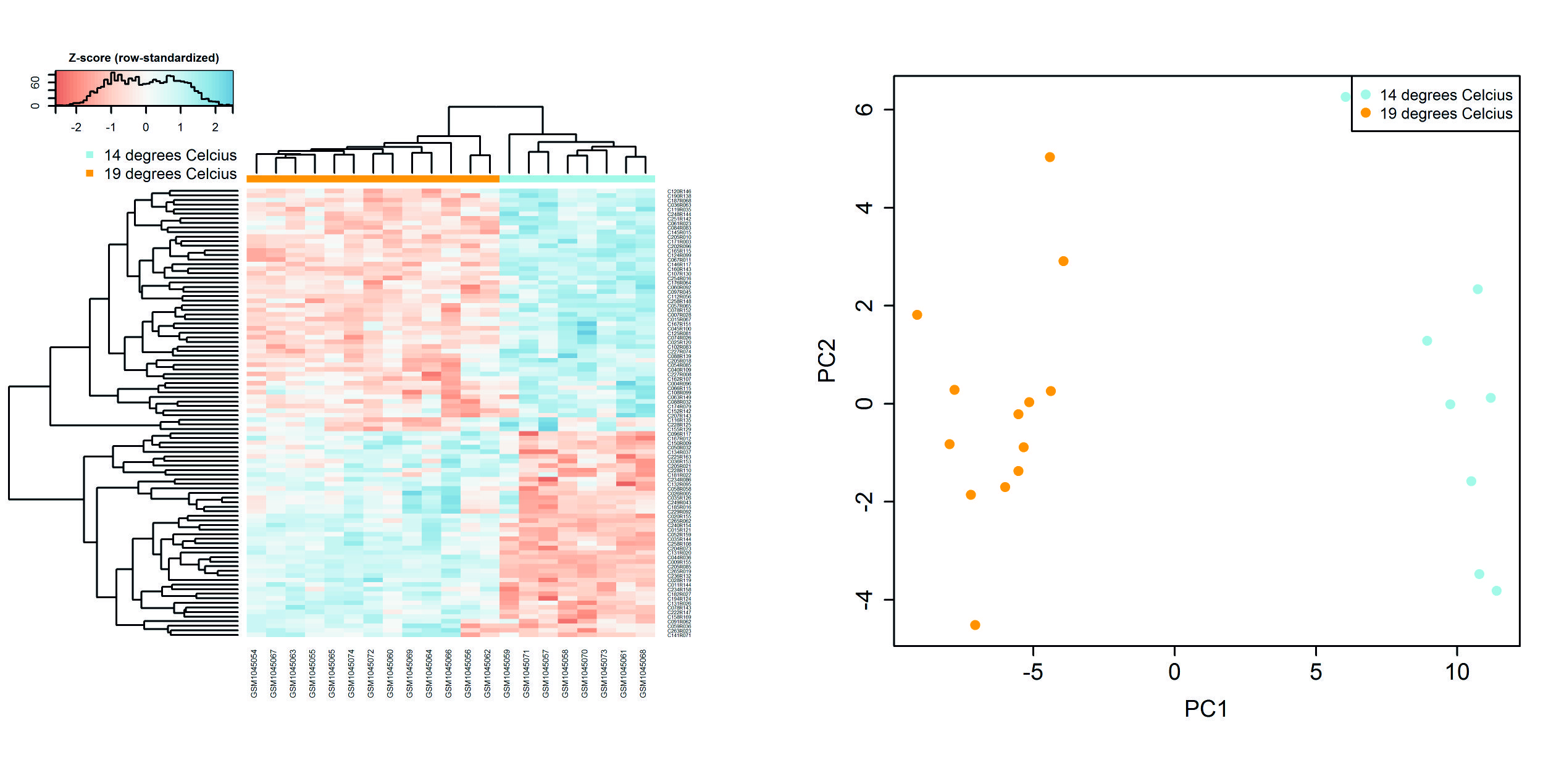

Supplement: Supplementary file 4 — Figure S3. Shown is a heatmap (left) and PCA plot (right) of 21 sockeye salmon samples (2007) for 98 features returned by Gene Shaving applied to the filtered 2007 sockeye salmon 30,072-feature data set. (JPG 2470 kb) [file 12864_2018_5108_MOESM4_ESM.jpg]

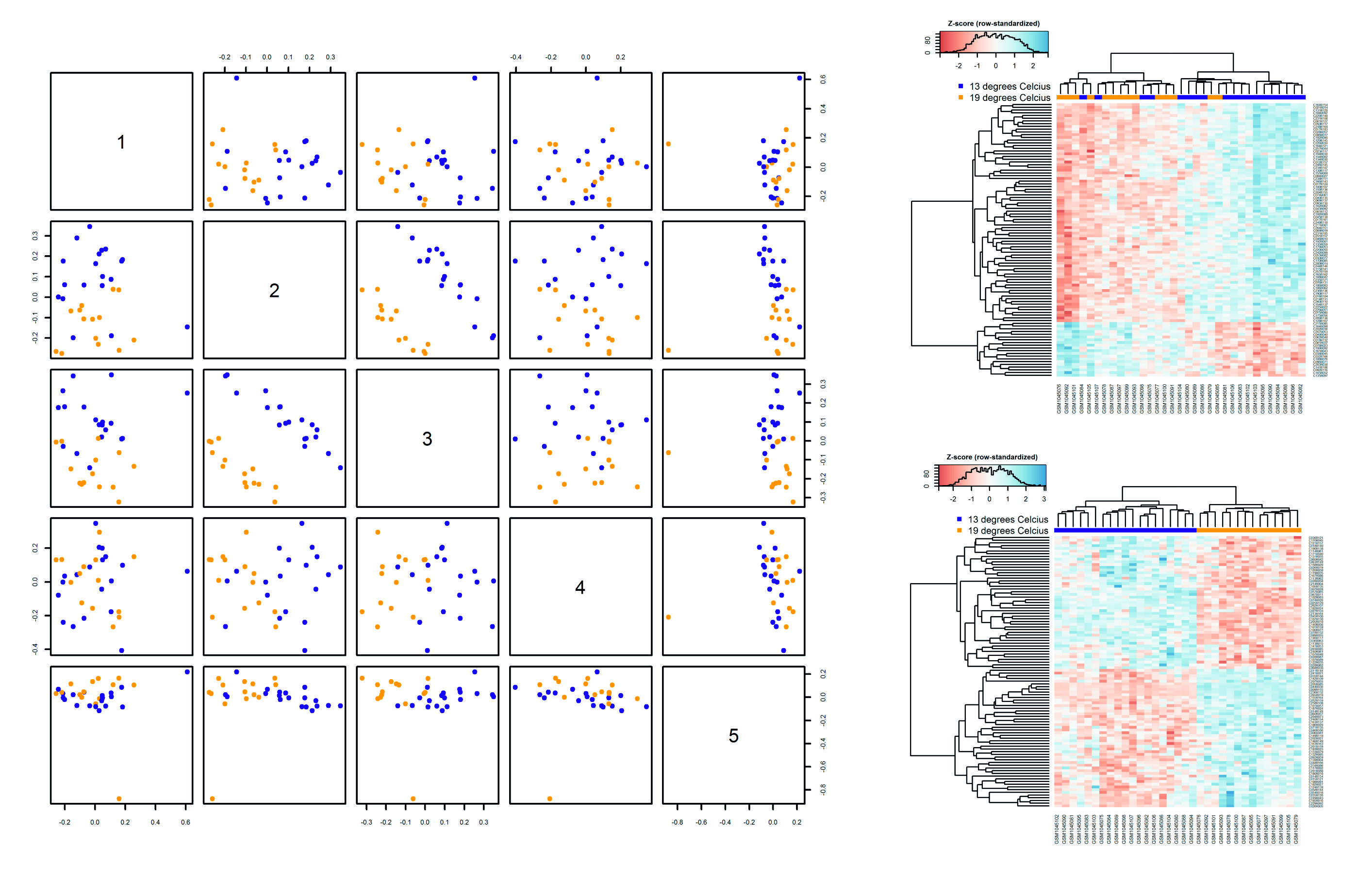

Supplement: Supplementary file 5 — Figure S4. Shown are sPCA scatter plots (left) and heatmaps for sPCA component 2 (top right) and component 3 (bottom right) for 33 sockeye salmon samples (2008). 100 features were returned for each of the sPCA components when sPCA was applied to the filtered 2008 sockeye salmon 29,657-feature data set. (JPG 3231 kb) [file 12864_2018_5108_MOESM5_ESM.jpg]
