# Supplementary material for: Developing specific molecular biomarkers for thermal stress in salmonids
Source: BMC Genomics. 2018 Oct 16;19:749. doi: 10.1186/s12864-018-5108-9 (PMC6192343; doi:10.1186/s12864-018-5108-9)
Supplement: Supplementary file 6 — Table S2. Gene summary table for 153 EST identifiers on cGRASP44K that define candidate temperature biomarker signature CTS001. The signature is mainly composed of 139 identifiers that were found in the intersection of robust limma analysis based on high and low temperature samples in the 2007, 2008 and 2009 MGL Temperature data set (CS0101i). Four additional features were found in Jeffries et al. 2014b published 49-identifier list and an additional ten EST identifiers were found in at least two of 3 year-based unsupervised exploratory analyses of the MGL 2007–2009 Temperature data. (DOC 183 kb) [file 12864_2018_5108_MOESM6_ESM.doc]

Table S2 Gene summary table for 153 EST identifiers on cGRASP44K that define candidate temperature biomarker signature CTS001. The signature is mainly composed of 139 identifiers that were found in the intersection of robust limma analysis based on high and low temperature samples in the 2007, 2008 and 2009 MGL Temperature data set (CS0101i). Four additional features were found in Jeffries et al. 2014b published 49-identifier list and an additional ten EST identifiers were found in at least two of three year-based unsupervised exploratory analyses of the MGL 2007-2009 Temperature data.

| **Feature** | **Gene ID** | **Gene Name** | **Combined Temperature Intersect 2007-2009 (CS0101i)** | **Jeffries Combined Temperature Intersect 2007-2009 (ES0013)** | **Pink Temperature EX0101a** | **2007 Sockeye Temperature EX0102a** | **2008 Sockeye Temperature EX103a** |
| --- | --- | --- | --- | --- | --- | --- | --- |
| C044R036 | SERPINH1 | Serpin H1 precursor | X | X | X | X | X |
| C265R019 | SERPINH1 | Serpin H1 precursor | X | X | X | X | X |
| C009R155 | SERPINH1 | Serpin H1 precursor | X | X | X | X | X |
| C205R085 | SERPINH1 | Serpin H1 precursor | X | X | X | X | X |
| C107R130 | CIRBP | Cold-inducible RNA-binding protein | X | X | X | X | X |
| C236R132 | SERPINH1 | Serpin H1 precursor | X | X | X | X | X |
| C025R120 | SFRS 9 | Splicing factor, arginine/serine-rich 9 | X | X | X | X | X |
| C074R026 | SFRS 9 | Splicing factor, arginine/serine-rich 9 | X | X | X | X | X |
| C078R152 | SFRS2 | Splicing factor, arginine/serine-rich 2 | X | X | X | X | X |
| C020R155 | HSP90ab1 | Heat shock protein HSP 90-beta | X | X | X | X |  |
| C187R068 | SFRS2 | Splicing factor, arginine/serine-rich 2 | X | X | X | X |  |
| C254R016 | SFRS2 | Splicing factor, arginine/serine-rich 2 | X | X | X | X |  |
| C096R117 | EIF4A2 | Eukaryotic initiation factor 4A-II | X | X | X | X |  |
| C140R056 | atp1a1 | Sodium/potassium-transporting ATPase subunit alpha-1 precursor | X | X | X |  | X |
| C067R011 | FKBP10 | FK506-binding protein 10 precursor | X | X |  | X | X |
| C045R100 | atp1a1 | Sodium/potassium-transporting ATPase subunit alpha-1 precursor | X | X |  | X | X |
| C240R154 | Eif4enif1 | Eukaryotic translation initiation factor 4E transporter | X | X |  | X | X |
| C007R028 | MPDU1 | Mannose-P-dolichol utilization defect 1 protein | X | X |  | X | X |
| C205R010 | SCFD1 | Sec1 family domain-containing protein 1 | X | X |  | X | X |
| C036R063 | SFRS2 | Splicing factor, arginine/serine-rich 2 | X | X |  | X | X |
| C035R144 | SEPW1 | Selenoprotein W | X | X |  | X | X |
| C258R108 | SEPW1 | Selenoprotein W | X | X |  | X | X |
| C182R027 | HSP90AA1 | Heat shock protein HSP 90-alpha | X | X |  | X | X |
| C204R073 | ZBTB46 | Zinc finger and BTB domain-containing protein 46 | X | X |  | X | X |
| C102R083 | EEF2 | Elongation factor 2 | X | X |  | X | X |
| C052R159 | SEPW1 | Selenoprotein W | X | X |  | X | X |
| C131R020 | RPL32 | 60S ribosomal protein L32 | X | X |  | X |  |
| C165R115 | FKBP10 | FK506-binding protein 10 precursor | X | X |  | X |  |
| C124R099 | FKBP10 | FK506-binding protein 10 precursor | X | X |  | X |  |
| C158R169 | Rpl36a | 60S ribosomal protein L36a | X | X |  | X |  |
| C160R143 | zgc:63572 | Transmembrane protein 185-like | X | X |  | X |  |
| C171R003 | FKBP10 | FK506-binding protein 10 precursor | X | X |  | X |  |
| C097R045 | carm1 | Histone-arginine methyltransferase CARM1 | X | X |  | X |  |
| C061R023 | AP3S1 | AP-3 complex subunit sigma-1 | X | X |  | X |  |
| C125R081 | IDH3B | Isocitrate dehydrogenase [NAD] subunit beta, mitochondrial precursor | X | X |  | X |  |
| C084R083 | SFRS2 | Splicing factor, arginine/serine-rich 2 | X | X |  | X |  |
| C014R149 | Map3k14 | Mitogen-activated protein kinase kinase kinase 14 | X | X |  |  | X |
| C155R164 | SEPW1 | Selenoprotein W | X | X |  |  | X |
| C241R021 | ST6GALNAC6 | Alpha-N-acetylgalactosaminide alpha-2,6-sialyltransferase 6 | X | X |  |  | X |
| C182R024 |  | UNKNOWN | X | X |  |  | X |
| C154R080 | FKBP10 | FK506-binding protein 10 precursor | X | X |  |  |  |
| C194R063 | CRELD1 | Cysteine-rich with EGF-like domain protein 1 precursor | X | X |  |  |  |
| C128R089 | athl1 | Acid trehalase-like protein 1 | X | X |  |  |  |
| C246R153 | park7 | Protein DJ-1 | X | X |  |  |  |
| C102R089 | KCT2 | Keratinocytes-associated transmembrane protein 2 precursor | X | X |  |  |  |
| C190R138 | Zmynd11 | Zinc finger MYND domain-containing protein 11 | X |  | X | X | X |
| C026R101 | DNAJA4 | DnaJ homolog subfamily A member 4 | X |  | X |  |  |
| C026R122 | CIRBP | Cold-inducible RNA-binding protein | X |  | X |  |  |
| C057R065 | C18orf8 | Uncharacterized protein C18orf8 | X |  |  | X | X |
| C015R121 | HSP90AA1 | Heat shock protein HSP 90-alpha | X |  |  | X | X |
| C011R144 |  | Thymosin beta-a | X |  |  | X | X |
| C119R035 | Tcf12 | Transcription factor 12 | X |  |  | X | X |
| C054R085 |  | UNKNOWN | X |  |  | X |  |
| C202R096 | Brp44l | Brain protein 44-like protein | X |  |  | X |  |
| C088R032 | cuta | Protein CutA homolog precursor | X |  |  | X |  |
| C225R163 | CA6 | Carbonic anhydrase 6 precursor | X |  |  | X |  |
| C174R079 | SFRS2 | Splicing factor, arginine/serine-rich 2 | X |  |  | X |  |
| C015R067 | TMEM16A | Transmembrane protein 16A | X |  |  | X |  |
| C251R142 | CIRBP | Cold-inducible RNA-binding protein | X |  |  | X |  |
| C040R109 |  | UNKNOWN | X |  |  | X |  |
| C120R146 | HIF1A | Hypoxia-inducible factor 1 alpha | X |  |  | X |  |
| C066R115 | fdxr | NADPH:adrenodoxin oxidoreductase, mitochondrial precursor | X |  |  | X |  |
| C145R015 |  | Tubulin alpha chain, testis-specific | X |  |  | X |  |
| C134R037 | HSPA4L | Heat shock 70 kDa protein 4L | X |  |  | X |  |
| C050R032 | SFRS7 | Splicing factor, arginine/serine-rich 7 | X |  |  | X |  |
| C198R091 | HSP90a.1 | Heat shock protein HSP 90-alpha 1 | X |  |  |  | X |
| C142R109 | SEPW1 | Selenoprotein W | X |  |  |  | X |
| C199R092 |  | UNKNOWN | X |  |  |  | X |
| C046R166 | RBMX | Heterogeneous nuclear ribonucleoprotein G | X |  |  |  | X |
| C135R059 | WASF2 | Wiskott-Aldrich syndrome protein family member 2 | X |  |  |  | X |
| C164R154 | EXOC3L2 | Exocyst complex component 3-like protein 2 | X |  |  |  | X |
| C060R057 | MAP3K14 | Mitogen-activated protein kinase kinase kinase 14 | X |  |  |  | X |
| C018R140 | C24G6.8 | Probable peptidyl-tRNA hydrolase 2 | X |  |  |  | X |
| C244R146 | ndrg2 | Protein NDRG2 | X |  |  |  | X |
| C163R137 | Nek4 | Serine/threonine-protein kinase Nek4 | X |  |  |  | X |
| C078R023 | DEK | Protein DEK | X |  |  |  | X |
| C262R107 | EEF2 | Elongation factor 2 | X |  |  |  | X |
| C089R055 | UBE2Q2 | Ubiquitin-conjugating enzyme E2 Q2 | X |  |  |  | X |
| C092R059 | EIF4A2 | Eukaryotic initiation factor 4A-II | X |  |  |  | X |
| C101R159 | Tmed2 | Transmembrane emp24 domain-containing protein 2 precursor | X |  |  |  | X |
| C090R103 | Slc45a2 | Membrane-associated transporter protein | X |  |  |  |  |
| C169R048 | Hspa8 | Heat shock cognate 71 kDa protein | X |  |  |  |  |
| C217R107 |  | UNKNOWN | X |  |  |  |  |
| C222R071 | Slc45a2 | Membrane-associated transporter protein | X |  |  |  |  |
| C239R009 | Slc17a5 | Sialin | X |  |  |  |  |
| C174R067 | Anxa2 | Annexin A2 | X |  |  |  |  |
| C192R015 |  | UNKNOWN | X |  |  |  |  |
| C037R148 |  | UNKNOWN | X |  |  |  |  |
| C036R054 | Tuba1a | Tubulin alpha-1A chain | X |  |  |  |  |
| C240R152 |  | UNKNOWN | X |  |  |  |  |
| C193R155 | COX6B1 | Cytochrome c oxidase subunit VIb isoform 1 | X |  |  |  |  |
| C020R006 |  | Lysozyme g | X |  |  |  |  |
| C097R152 | GPS2 | G protein pathway suppressor 2 | X |  |  |  |  |
| C221R165 | mfsd7 | Major facilitator superfamily domain-containing protein 7 | X |  |  |  |  |
| C031R011 | SFRS7 | Splicing factor, arginine/serine-rich 7 | X |  |  |  |  |
| C070R119 | FKBP10 | FK506-binding protein 10 precursor | X |  |  |  |  |
| C113R139 | gamt | Guanidinoacetate N-methyltransferase | X |  |  |  |  |
| C062R114 | Tuba1a | Tubulin alpha-1A chain | X |  |  |  |  |
| C131R114 | PYGM | Glycogen phosphorylase, muscle form | X |  |  |  |  |
| C185R026 | RTCD1 | RNA 3'-terminal phosphate cyclase | X |  |  |  |  |
| C051R015 |  | Granzyme-like protein 1 precursor | X |  |  |  |  |
| C242R131 | CBX1 | Chromobox protein homolog 1 | X |  |  |  |  |
| C101R077 | SERPINH1 | Serpin H1 precursor | X |  |  |  |  |
| C071R163 | SFRS7 | Splicing factor, arginine/serine-rich 7 | X |  |  |  |  |
| C088R019 |  | Histone H2A | X |  |  |  |  |
| C072R039 |  | UNKNOWN | X |  |  |  |  |
| C230R135 | ATP6V0C | Vacuolar ATP synthase 16 kDa proteolipid subunit | X |  |  |  |  |
| C142R150 | clptm1 | Cleft lip and palate transmembrane protein 1 homolog | X |  |  |  |  |
| C159R065 | NDUFS7 | NADH dehydrogenase [ubiquinone] iron-sulfur protein 7, mitochondrial precursor | X |  |  |  |  |
| C014R004 | FKBP10 | FK506-binding protein 10 precursor | X |  |  |  |  |
| C107R106 | SFRS1 | Splicing factor, arginine/serine-rich 1 | X |  |  |  |  |
| C051R114 |  | UNKNOWN | X |  |  |  |  |
| C213R047 | Glyat | Glycine N-acyltransferase | X |  |  |  |  |
| C057R105 |  | UNKNOWN | X |  |  |  |  |
| C263R094 | PDIA4 | Protein disulfide-isomerase A4 precursor | X |  |  |  |  |
| C117R126 |  | UNKNOWN | X |  |  |  |  |
| C142R097 | TNNC1 | Troponin C, slow skeletal and cardiac muscles | X |  |  |  |  |
| C187R026 | SFRS1 | Splicing factor, arginine/serine-rich 1 | X |  |  |  |  |
| C101R112 | HSP90ab1 | Heat shock protein HSP 90-beta | X |  |  |  |  |
| C018R129 | ALAD | Delta-aminolevulinic acid dehydratase | X |  |  |  |  |
| C119R133 | PEBP1 | Phosphatidylethanolamine-binding protein 1 | X |  |  |  |  |
| C074R053 | TNNC1 | Troponin C, slow skeletal and cardiac muscles | X |  |  |  |  |
| C114R035 | SFRS6 | Splicing factor, arginine/serine-rich 6 | X |  |  |  |  |
| C075R011 | Tuba1a | Tubulin alpha-1A chain | X |  |  |  |  |
| C135R033 | HCFC2 | Host cell factor 2 | X |  |  |  |  |
| C262R084 | PSME3 | Proteasome activator complex subunit 3 | X |  |  |  |  |
| C002R034 | TNNC1 | Troponin C, slow skeletal and cardiac muscles | X |  |  |  |  |
| C036R039 | Ehd1 | EH domain-containing protein 1 | X |  |  |  |  |
| C204R003 | bhlhb5 | Class B basic helix-loop-helix protein 5 | X |  |  |  |  |
| C025R140 | Dcun1d1 | DCN1-like protein 1 | X |  |  |  |  |
| C065R036 | acsf2 | Acyl-CoA synthetase family member 2, mitochondrial precursor | X |  |  |  |  |
| C260R050 |  | sialic acid acetylesterase | X |  |  |  |  |
| C257R028 | Stmn1 | Stathmin | X |  |  |  |  |
| C115R159 | OSBPL11 | Oxysterol-binding protein-related protein 11 | X |  |  |  |  |
| C084R135 | SFRS7 | Splicing factor, arginine/serine-rich 7 | X |  |  |  |  |
| C227R013 | Dph3 | DPH3 homolog | X |  |  |  |  |
| C058R073 | kctd6 | BTB/POZ domain-containing protein KCTD6 | X |  |  |  |  |
| C194R143 |  | UNKNOWN | X |  |  |  |  |
| C071R082 | CD226 | CD226 antigen precursor | X |  |  |  |  |
| C074R104 | COX6B1 | Cytochrome c oxidase subunit VIb isoform 1 |  | X |  |  |  |
| C042R086 | COX6B1 | Cytochrome c oxidase subunit VIb isoform 1 |  | X |  |  |  |
| C149R170 | COX6B1 | Cytochrome c oxidase subunit VIb isoform 1 |  | X |  |  |  |
| C190R095 | C1orf124 | Zinc finger RAD18 domain-containing protein C1orf124 |  | X |  |  |  |
| C059R036 | HSC71 | Heat shock cognate 70 kDa protein |  |  | X | X |  |
| C155R086 | EDEM1 | ER degradation-enhancing alpha-mannosidase-like 1 |  |  | X |  |  |
| C184R041 | PRMT2 | Protein arginine N-methyltransferase 2 |  |  | X |  |  |
| C196R078 | MPZL2 | Myelin protein zero-like protein 2 precursor |  |  | X |  |  |
| C026R005 |  | Heat shock 70 kDa protein 1 |  |  |  | X | X |
| C035R126 | HSP70 | Heat shock 70 kDa protein |  |  |  | X | X |
| C063R149 | CCDC16 | Coiled-coil domain-containing protein 16 |  |  |  | X | X |
| C132R095 |  | Uncharacterized protein C14orf24 homolog |  |  |  | X | X |
| C146R117 | NACA | Nascent polypeptide-associated complex subunit alpha |  |  |  | X | X |
| C185R016 | HSP70 | Heat shock 70 kDa protein |  |  |  | X | X |
| C229R092 | HSP70 | Heat shock 70 kDa protein |  |  |  | X | X |
| C234R086 | Camk1 | Calcium/calmodulin-dependent protein kinase type 1 |  |  |  | X | X |
| C249R043 | HSP70 | Heat shock 70 kDa protein |  |  |  | X | X |
